# Supplementary material for: A comprehensive molecular atlas of the mesenchymal cell types in the mouse liver
Source: EMBO Rep. 2025 Sep 15;26(21):5326–59. doi: 10.1038/s44319-025-00580-9 (PMC12592516; doi:10.1038/s44319-025-00580-9)
Supplement: Supplementary file 1 — Appendix [file 44319_2025_580_MOESM1_ESM.pdf]

Appendix document for

A comprehensive molecular atlas of the mesenchymal cell types in the mouse liver.

Pietilä *et al.*

Table of Content:

Appendix Figure S1	Page 1
Appendix Figure S2	Page 3
Appendix Figure S3	Page 4
Appendix Figure S4	Page 5
Appendix Figure S5	Page 7
Appendix Figure S6	Page 9
Appendix Figure S7	Page 11
Appendix Figure S8	Page 13
References	Page 14

Appendix Figure S1

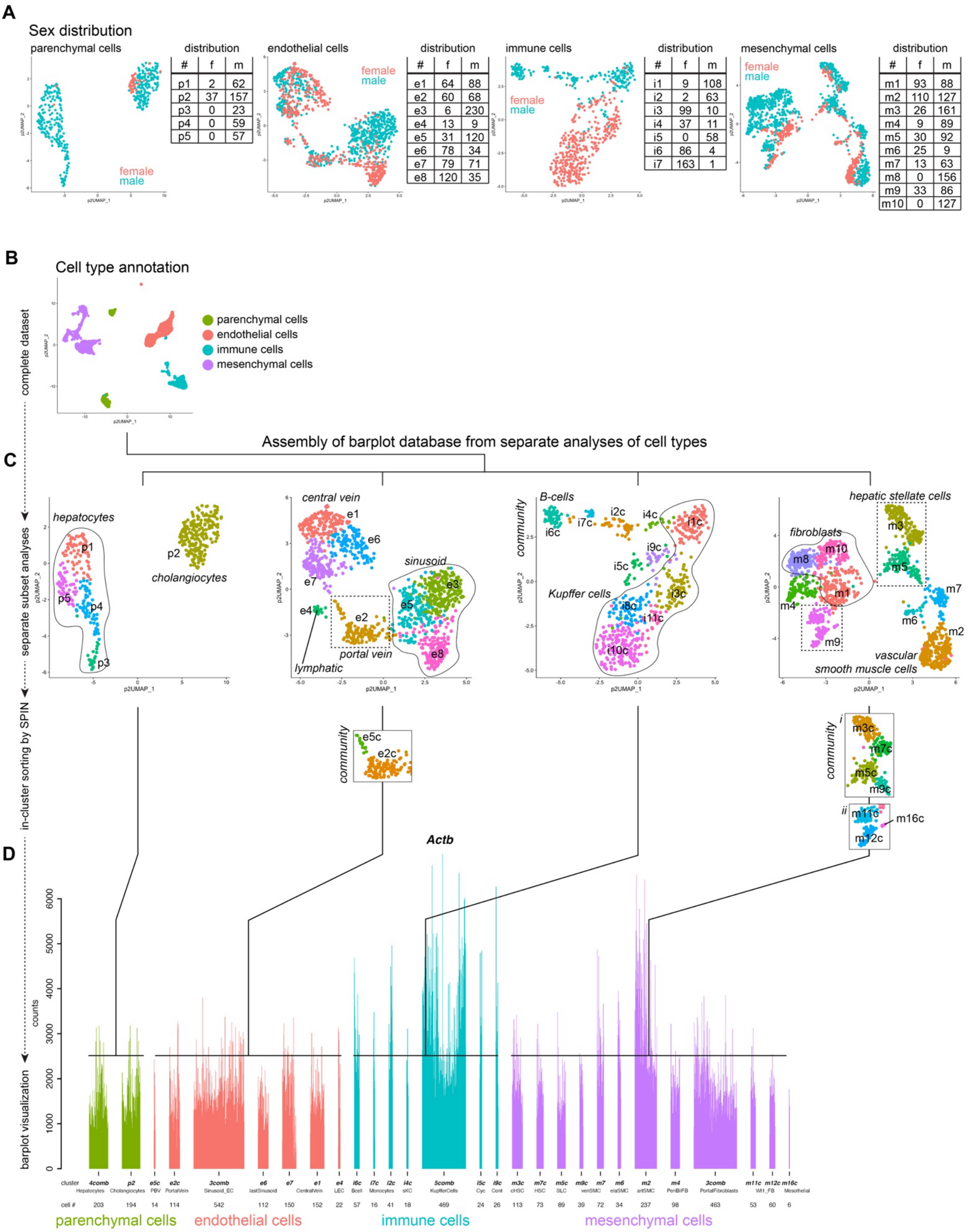

#	f	m
p1	2	62
p2	37	157
p3	0	23
p4	0	59
p5	0	57

#	f	m
e1	64	88
e2	60	68
e3	6	230
e4	13	9
e5	31	120
e6	78	34
e7	79	71
e8	120	35

#	f	m
i1	9	108
i2	2	63
i3	99	10
i4	37	11
i5	0	58
i6	86	4
i7	163	1

#	f	m
m1	93	88
m2	110	127
m3	26	161
m4	9	89
m5	30	92
m6	25	9
m7	13	63
m8	0	156
m9	33	86
m10	0	127

A UMAP visualization for all separately analyzed subsets (left to right: parenchymal cells, endothelial cells, immune cells, mesenchymal cells), color coded according to sex of the source

mouse for cell isolation. The cell distribution between female (f) and male (m) in the identified clusters (pagoda2 multilevel) is given in the accompanying tables. **B-D** Overview of the construction of the barplot visualization accessible at https://muhldatahub.org/Publications/LiverScRNAseq/database.html. **B** UMAP visualization of the complete dataset color coded for the identified cell type classes. **C** UMAP visualization color coded for the clustering results for the separately analyzed subsets (left to right: parenchymal cells, endothelial cells, immune cells, mesenchymal cells). If clusters identified by the high-resolution community setting were used, these are indicated by boxed areas and in the crop-outs shown below the main UMAP plots. Clusters that were combined for the barplot visualization are encircled with solid lines and indicated in the barplot by ‘comb’ (combined) annotation. **D** The barplot visualization showing the expression level of *Actb* as displayed in the online database. The bars are color coded according to cell class subset. Each bar represents a single-cell transcriptome with fixed x-axis position. Of note, the cell distribution within each barplot cluster has been reordered using the SPIN algorithm.

Appendix Figure S2

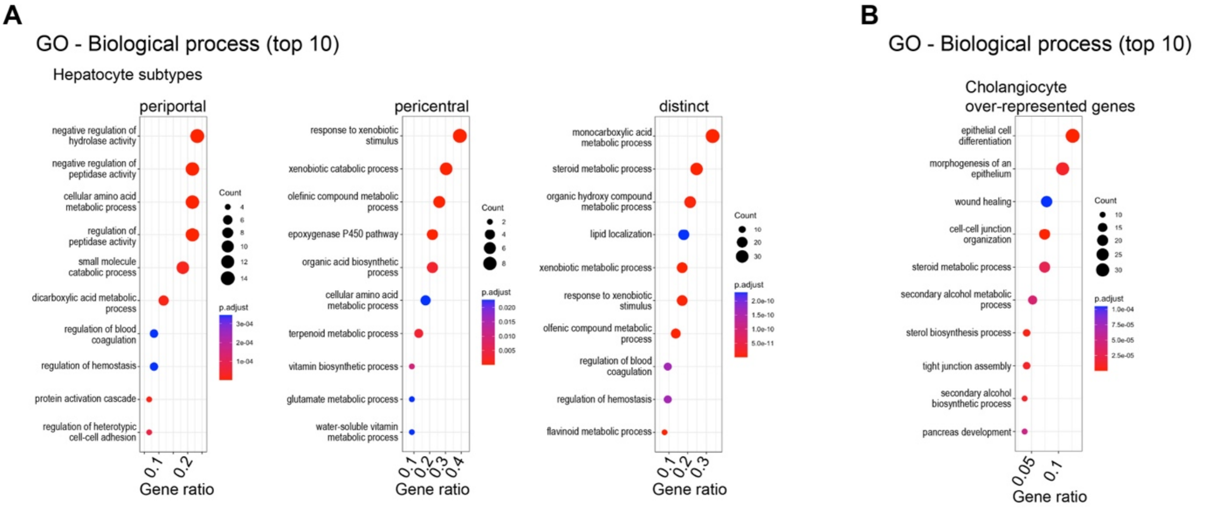

A Dot plots showing the top10 overrepresented GO terms for the different hepatocyte subpopulations (left to right: periportal, pericentral, distinct). **B** Dot plot showing the top10 overrepresented GO terms for genes with enriched expression in cholangiocytes when compared to all other cells in the complete dataset.

Appendix Figure S3

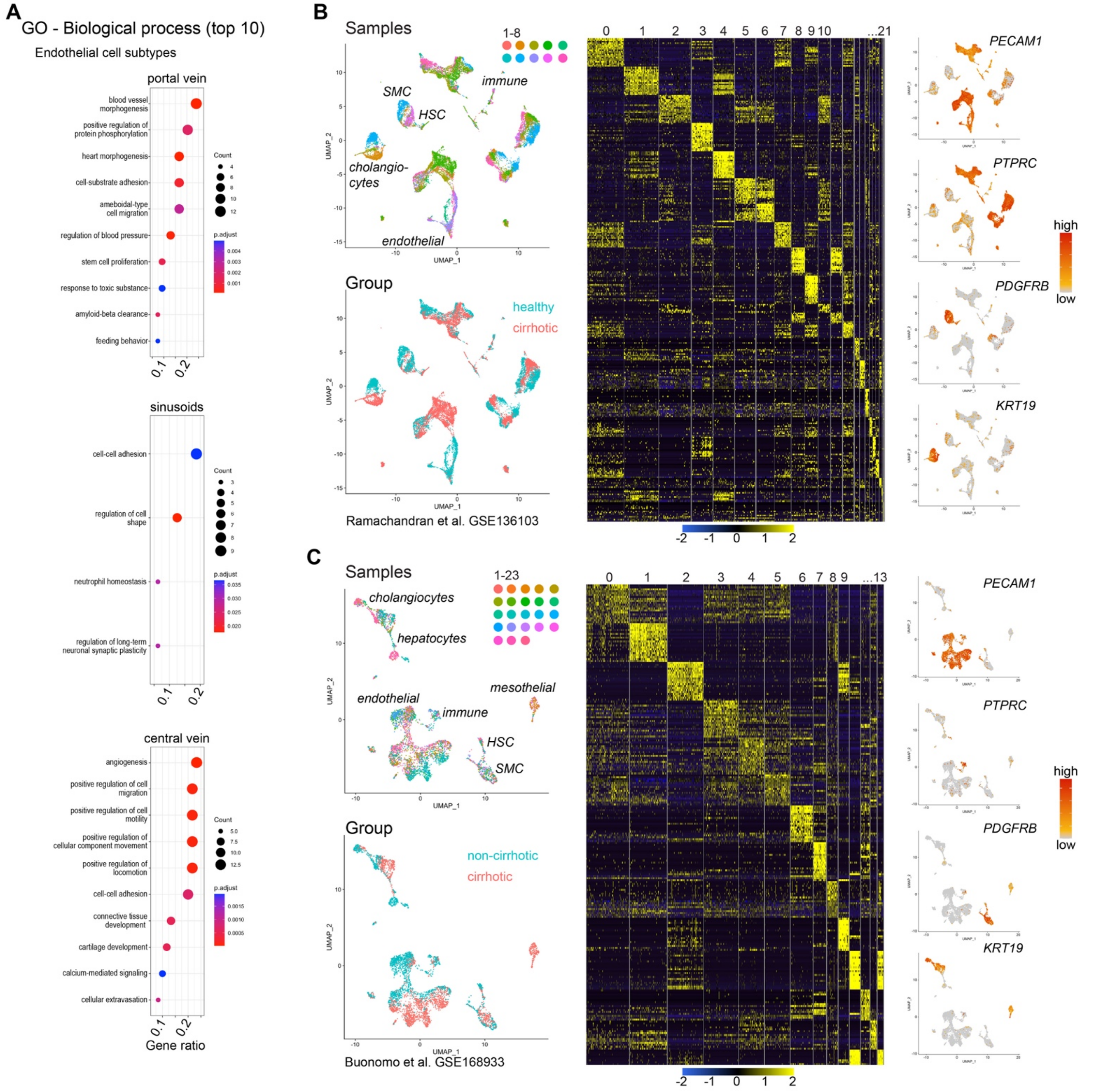

A Dot plots showing the top10 overrepresented GO terms for genes enriched in endothelial cell subpopulations (top to bottom: portal vein, sinusoids, central vein). **B, C** Analysis of the GSE136103 human liver single-cell RNA-sequencing dataset (Data ref: Ramachandran *et al*, 2019) (B), and the GSE168933 dataset (Data ref: Buonomo *et al*, 2022) (C) using the Seurat R-software package. UMAP visualization color coded for the respective different samples (left, upper panel) or the disease group (left, lower panel). A heat map showing the expression level of the top20 representative genes for each identified cluster in the respective dataset (middle panel), and UMAP visualization of the expression level of cell class markers *PECAM1* (endothelial cells), *PTPRC* (immune cells), *PDGFRB* (mesenchymal cells), and *KRT19* (epithelial cells) (right panel).

Appendix Figure S4

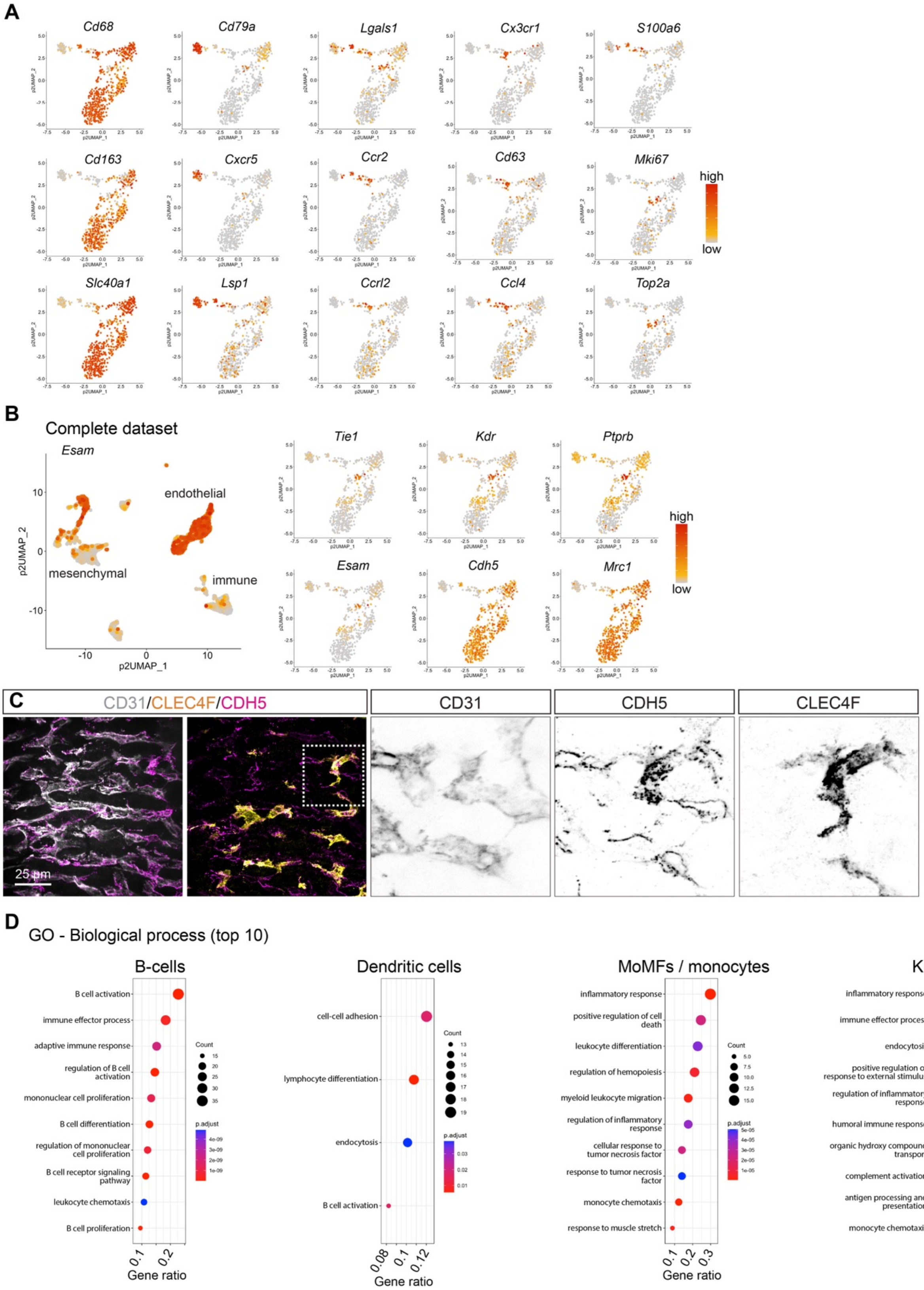

A UMAP visualization of the expression level of immune cell marker genes in the immune cell subset. **B** UMAP visualization of the expression level of *Esam* in the complete dataset (left panel) and UMAP visualization of endothelial cell marker genes (*Tie1*, *Kdr*, *Ptprb*) and the Kupffer cell marker (*Mrc1*) together with *Esam* and *Cdh5* in the immune cell subset (right panel). **C** IF for

CD31, CLEC4F, and CDH5 on a liver tissue section. **D** Dot plots showing the top10 GO terms overrepresented for genes with enriched expression in the different identified immune cell populations, (from left to right) B-cells, myeloid monocytic/dendritic cells, MoMFs, or Kupffer cells.

Appendix Figure S5

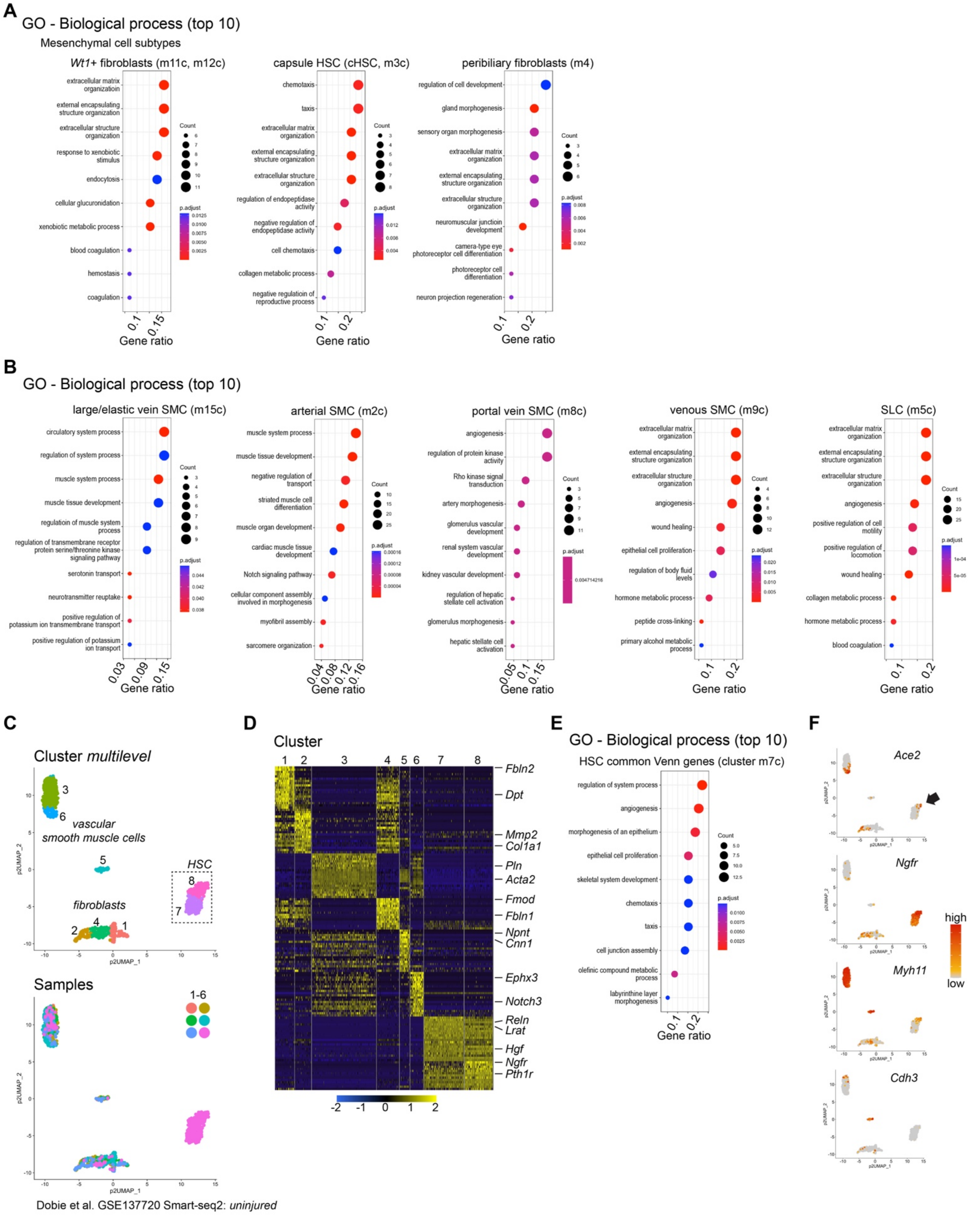

A Dot plots showing the top10 overrepresented GO terms for genes with enriched expression in *Wt1*+ fibroblasts (left panel), cHSC (middle panel), or peribiliary fibroblasts (right panel). **B** Dot plots showing the top10 overrepresented GO terms for genes with enriched expression in the

different vascular mural cell subpopulations, (from left to right) large/elastic vein SMC, arterial SMC, portal vein SMC, venous SMC, and SLC. **C** UMAP visualization of the clustering results of the GSE137720 dataset (Data ref: Dobie *et al*, 2019), using cells from the uninjured samples (upper panel) and color coded for the six different samples (lower panel). **D** Heat map showing the expression level of the top20 identified genes for each cluster. **E** Dot plot showing the top10 overrepresented GO terms for genes with enriched expression in HSC (cluster #m7c), identified from the Venn analysis shown in Figure EV5E. **F** UMAP visualization of the expression level of HSC subtype markers, *Ace2*, *Ngfr*, *Cdh3* and *Myh11* in the GSE137720 dataset.

Appendix Figure S6

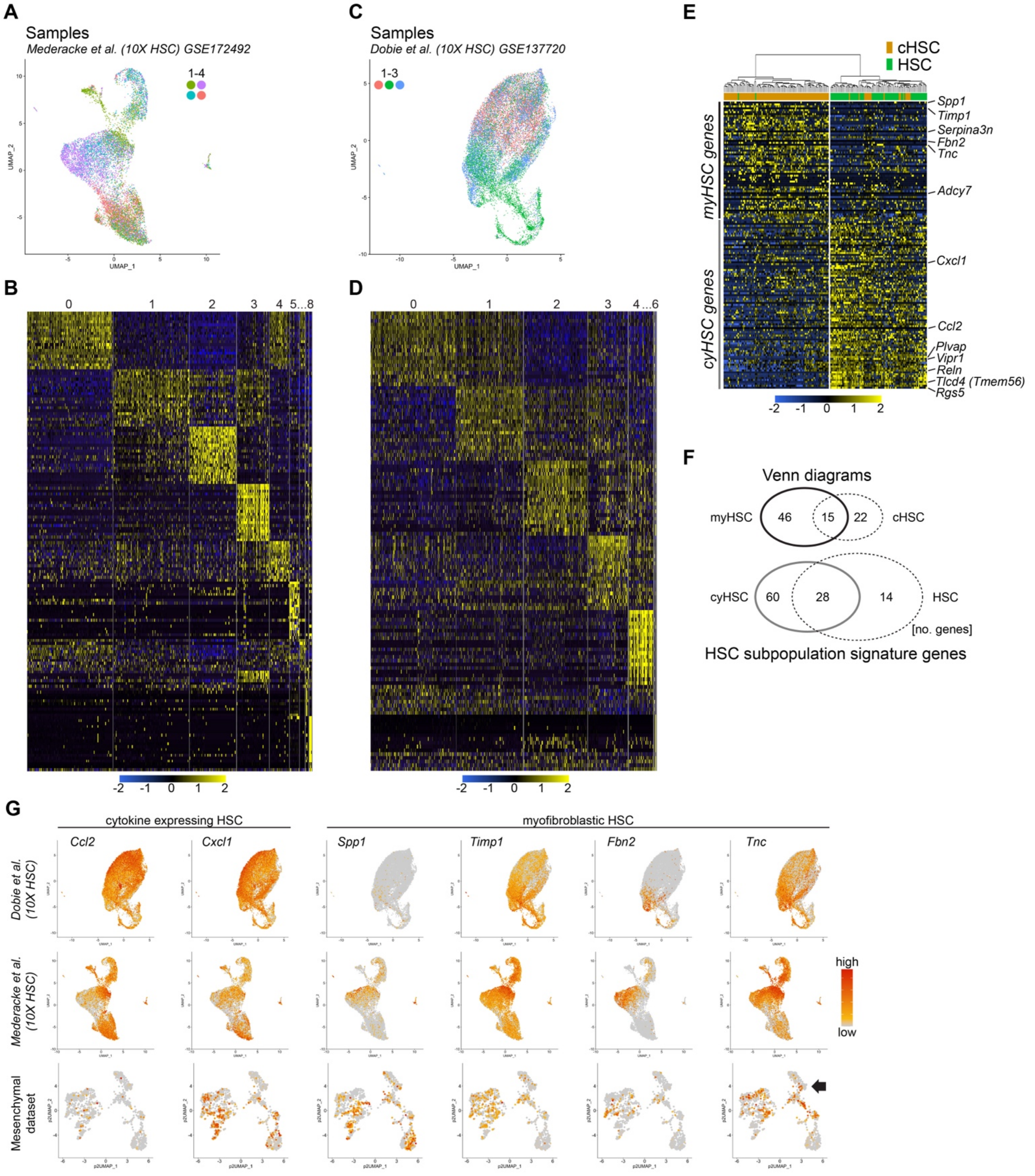

A UMAP visualization of the GSE172492 dataset (Data ref: Mederacke *et al*, 2022), color coded for the four different samples. Note that the dataset includes samples specifically enriched for HSC from the healthy and CCl_4 treated animals. **B** Heat map showing the expression level of the top20 representative genes for each identified cluster, determined using the Seurat R-software package. **C** UMAP visualization of the GSE137720 (10X) dataset (Data ref: Dobie *et al.*, 2019), color coded for the three different samples. Note that the dataset includes samples specifically

enriched for HSC from the uninjured and CCl_4 treated animals. **D** Heat map showing the expression level of the top20 representative genes for each identified cluster, determined using the Seurat R-software package. **E** Heat map showing the expression level of the top genes defined for myHSC or cyHSC, respectively (Filliol *et al*, 2022), in cHSC and HSC. Genes with average log-fold ≥1 were selected from the original Supplementary Tables 2 and 3 (Filliol *et al.*, 2022). Cells were clustered dependent on their expression pattern of the selected genes, using the pheatmap() function in R-software with clustering_method = “ward.D2”. **F** Venn diagrams showing the quantitative overlap of top genes defined for myHSC and cHSC (upper panel) or cyHSC and HSC (lower panel), respectively (Dataset EV2). Compare to Figure 8D for cHSC and HSC genes, and E for myHSC and cyHSC genes. **G** UMAP visualization of the expression level of exemplary genes with increased expression in cyHSC in response to CCl_4 challenge (*Ccl2* and *Cxcl1*), or genes with increased expression in myHSC in response to CCl_4 challenge (*Spp1*, *Timp1*, *Fbn2*, and *Tnc*) in the GSE172492 dataset (upper row) or the GSE137720 dataset (middle row). Note the lack of expression of these genes in the HSC clusters of the mesenchymal cell subset (lower row).

Appendix Figure S7

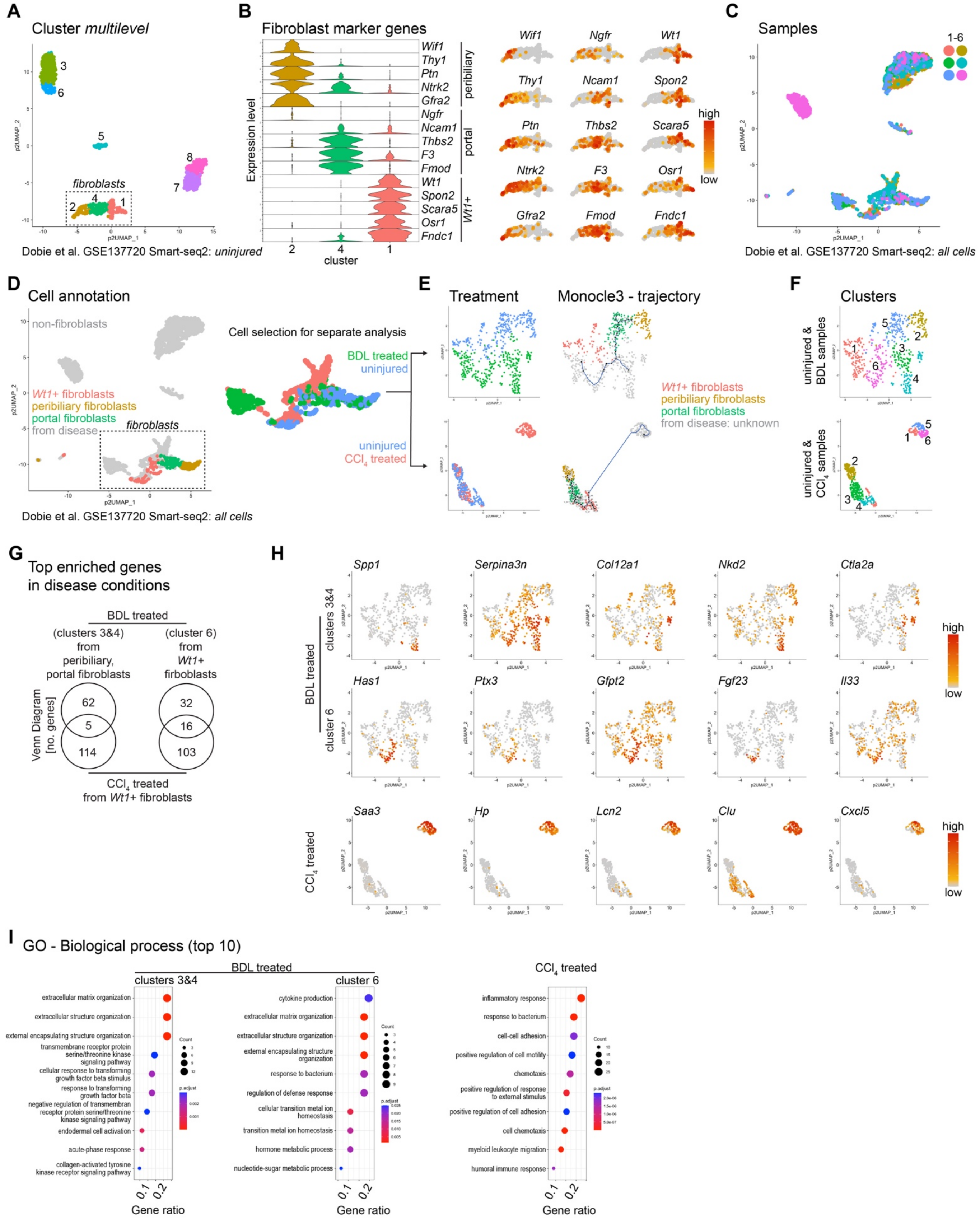

A UMAP visualization of the clustering result of the GSE137720 (Smart-seq2) dataset using cells from the uninjured samples (Data ref: Dobie *et al.*, 2019). Clusters identified as fibroblasts are indicated by the boxed area. **B** Violin plot showing the expression level of herein defined fibroblast

subset marker genes, in the three clusters of fibroblasts (left panel, n refers to cells: 2=76 , 4=104, 1=90) and UMAP visualization of the expression level of fibroblast marker genes in the magnified area (A) representing fibroblast clusters of the GSE137720 (Smart-seq2) dataset. **C** UMAP visualization of the GSE137720 (Smart-seq2) dataset, color coded for the six samples, using cells from all conditions (uninjured, bile duct ligation [BDL], and CCl_4 treated). **D** UMAP visualization of the GSE137720 (Smart-seq2) dataset color coded for the implied fibroblast subpopulation (compare to B). Note that only cells from the uninjured samples are colored according to their suggested fibroblast subpopulation (left panel). Magnified area containing fibroblast clusters of the UMAP visualization of the GSE137720 (Smart-seq2) dataset with all cells included, color coded for the disease groups (uninjured, BDL, CCl_4 treatment). **E** UMAP visualizations of the selected fibroblast subpopulations (upper panel: uninjured and BDL / lower panel: uninjured and CCl_4 treated), color coded for the disease group (left panel) or the fibroblast annotation together with the monocle3-calculated trajectory (right panel). **F** UMAP visualization of the selected fibroblast subpopulation analyses, color coded for the clustering results from pagoda2 (upper panel: uninjured and BDL / lower panel: uninjured and CCl_4 treated). **G** Venn diagrams showing the quantitative overlap of genes identified to be overexpressed in the different clusters with high pseudotime values from the disease models (Dataset EV3). Note the relatively low overlap of genes with increased expression between the disease models (BDL and CCl_4 treatment), suggesting distinct transcriptional programs activated between the two disease models and in distinct fibroblast subpopulations. **H** UMAP visualizations of the expression level of exemplary genes with increased expression in BDL (upper and middle row) or CCl_4 treated (lower row) fibroblast subpopulations. **I** Dot plots showing the top10 enriched GO terms from genes with increased expression in the different fibroblast subpopulations dependent on the disease model (from left to right: BDL clusters #3 and 4, BDL cluster #6, CCl_4 clusters #1,5,6).

Appendix Figure S8

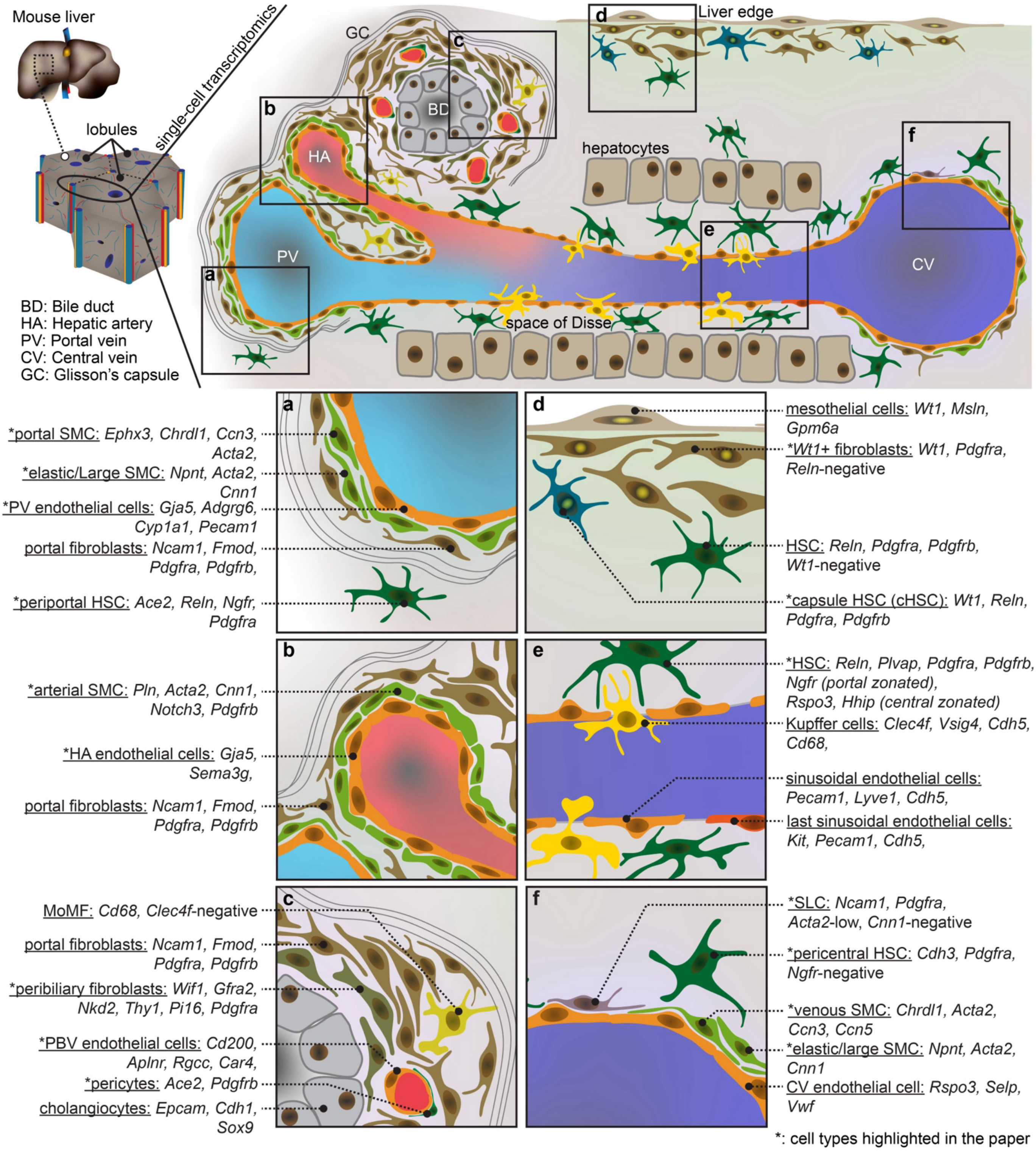

Summary figure depicting the liver cell types characterized in this study.

References

Buonomo EL, Mei S, Guinn SR, Leo IR, Peluso MJ, Nolan MA, Schildberg FA, Zhao L, Lian C, Xu S *et al* (2022) Gene Expression Omnibus GSE168933 (https://www.ncbi.nlm.nih.gov/geo/query/acc.cgi?acc=GSE168933) [DATASET]

Dobie R, Wilson-Kanamori JR, Henderson BEP, Smith JR, Matchett KP, Portman JR, Wallenborg K, Picelli S, Zagorska A, Pendem SV *et al* (2019) Gene Expression Omnibus GSE137720 (https://www.ncbi.nlm.nih.gov/geo/query/acc.cgi?acc=GSE137720) [DATASET]

Filliol A, Saito Y, Nair A, Dapito DH, Yu LX, Ravichandra A, Bhattacharjee S, Affo S, Fujiwara N, Su H *et al* (2022) Opposing roles of hepatic stellate cell subpopulations in hepatocarcinogenesis. *Nature* 610: 356-365

Mederacke I, Filliol A, Affo S, Nair A, Hernandez C, Sun Q, Hamberger F, Brundu F, Chen Y, Ravichandra A *et al* (2022) Gene Expression Omnibus GSE172492 (https://www.ncbi.nlm.nih.gov/geo/query/acc.cgi?acc=GSE172492) [DATASET]

Ramachandran P, Dobie R, Wilson-Kanamori JR, Dora EF, Henderson BEP, Luu NT, Portman JR, Matchett KP, Brice M, Marwick JA *et al* (2019) Gene Expression Omnibus GSE136103 (https://www.ncbi.nlm.nih.gov/geo/query/acc.cgi?acc=GSE136103) [DATASET]
