# Supplementary figures and images for: A comprehensive molecular atlas of the mesenchymal cell types in the mouse liver
Source: EMBO Rep. 2025 Sep 15;26(21):5326–59. doi: 10.1038/s44319-025-00580-9 (PMC12592516; doi:10.1038/s44319-025-00580-9)

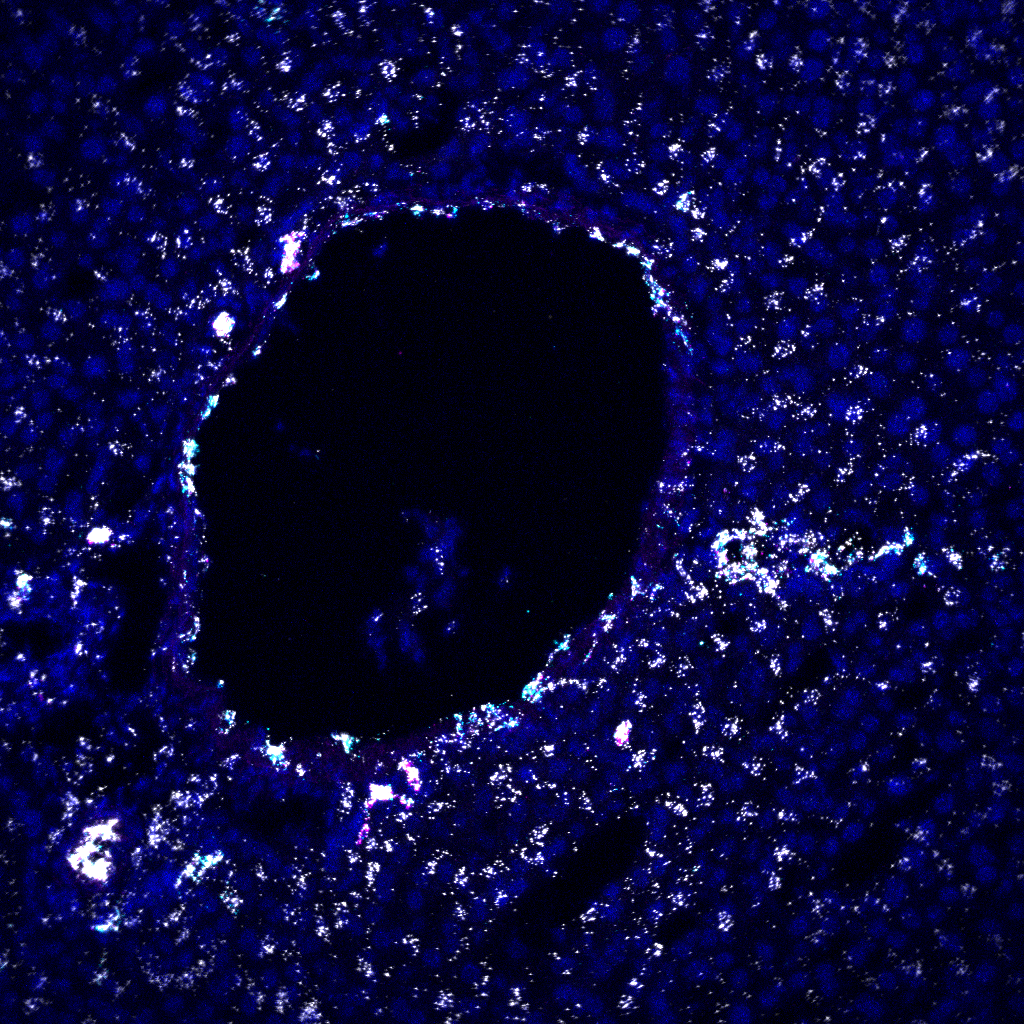

Supplement: Supplementary file 8 — Source data Fig. 2 [file 44319_2025_580_MOESM8_ESM.zip › Figure 2/2I/MAX_ISH_Nuclei_Gja5_Sema3g_Pecam1_portal_tract.tif]

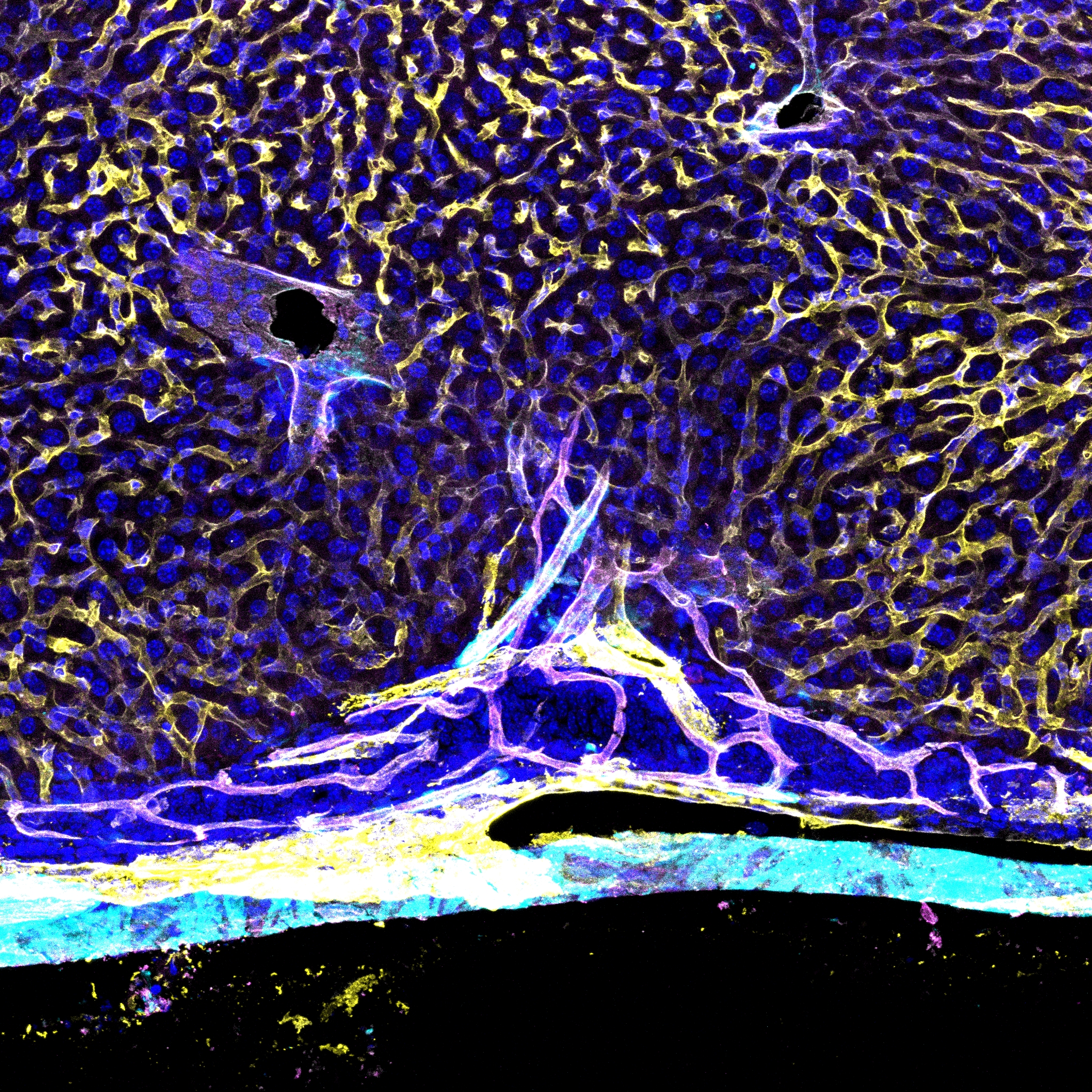

Supplement: Supplementary file 8 — Source data Fig. 2 [file 44319_2025_580_MOESM8_ESM.zip › Figure 2/2O/MAX_IF_Nuclei_CD31_Acta2GFP_LYVE1_CD200_portal_tract_bile_duct.jpg]

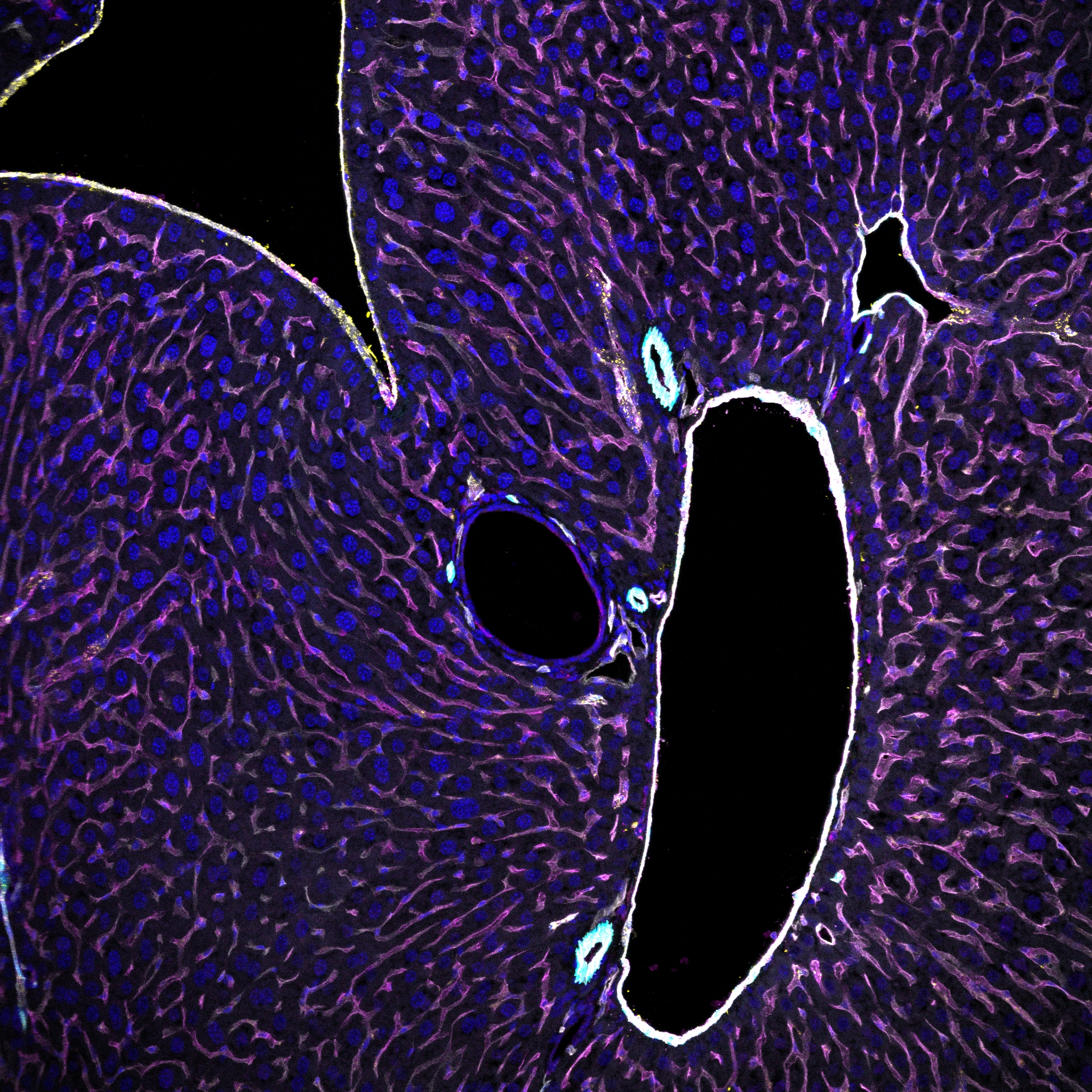

Supplement: Supplementary file 8 — Source data Fig. 2 [file 44319_2025_580_MOESM8_ESM.zip › Figure 2/2H/MAX_IF_Nuclei_CD31_Gja5GFP_VWF_CD36.jpg]

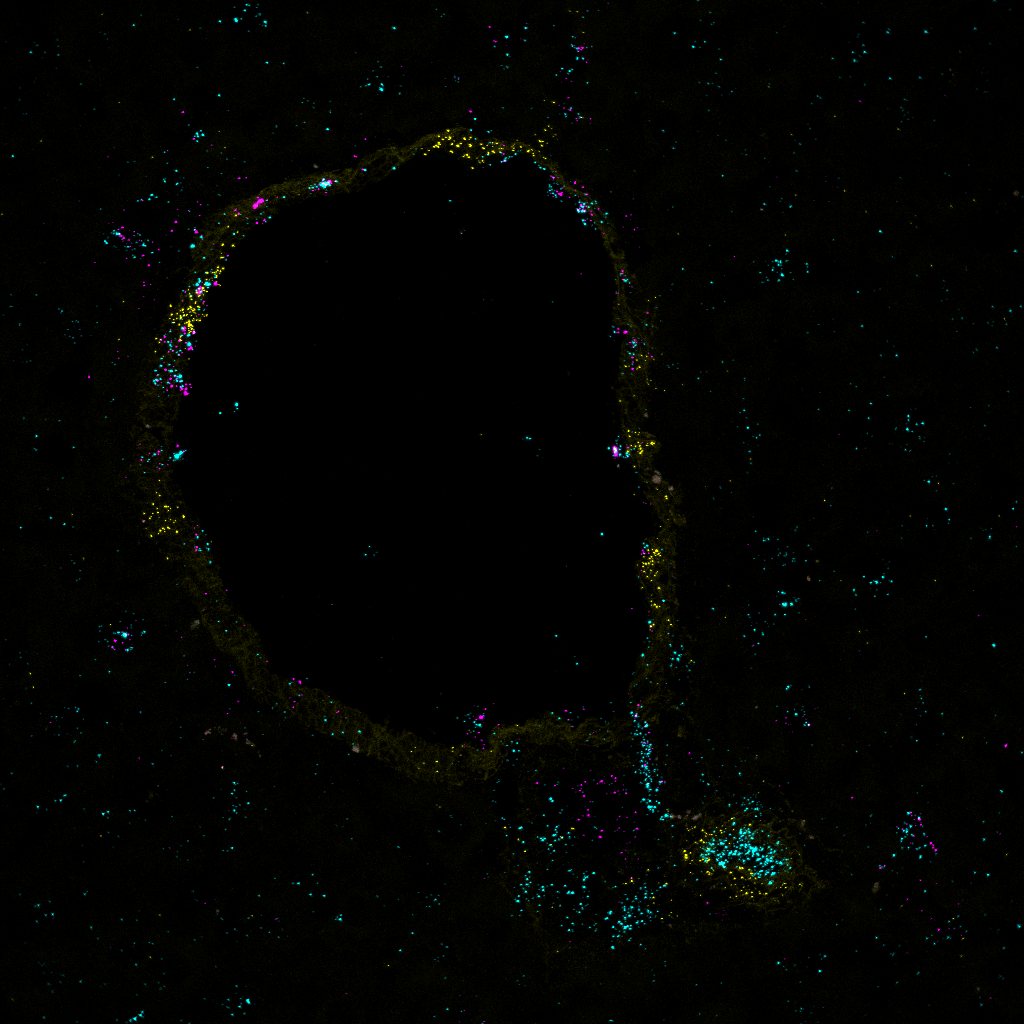

Supplement: Supplementary file 8 — Source data Fig. 2 [file 44319_2025_580_MOESM8_ESM.zip › Figure 2/2F/MAX_ISH_Pecam1_Adgrg6_Cnn1_portal_tract.tif]

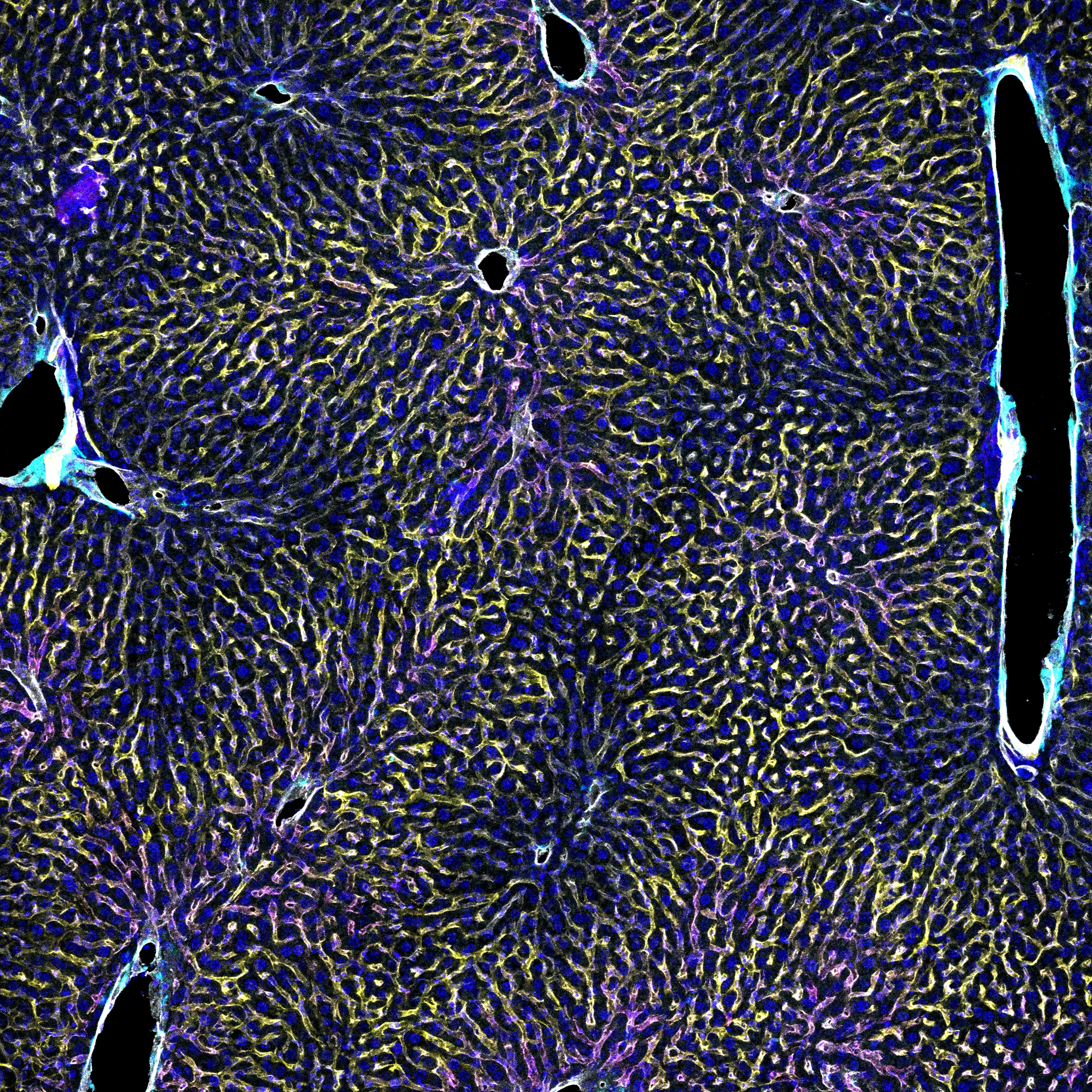

Supplement: Supplementary file 8 — Source data Fig. 2 [file 44319_2025_580_MOESM8_ESM.zip › Figure 2/2M/MAX_IF_Nuclei_CD31_Acta2GFP_LYVE1_KIT.jpg]

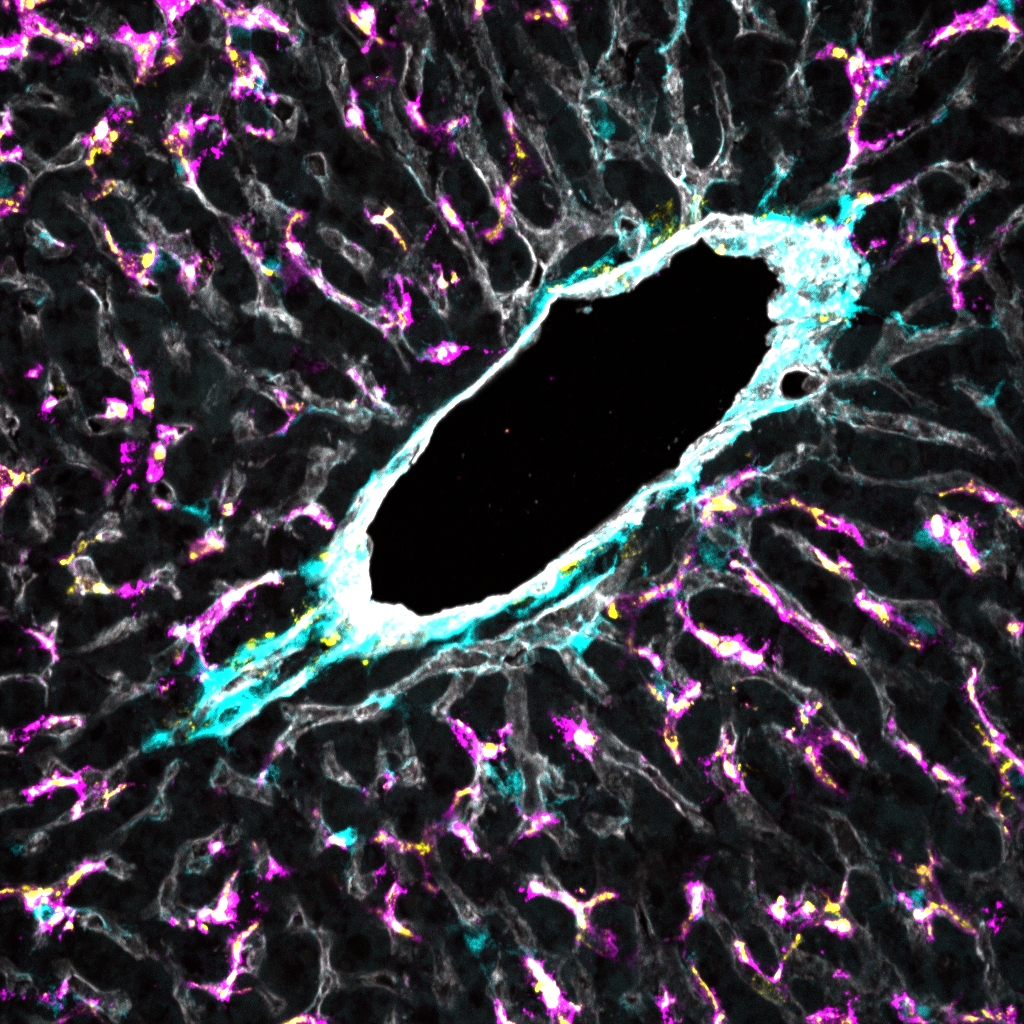

Supplement: Supplementary file 9 — Source data Fig. 3 [file 44319_2025_580_MOESM9_ESM.zip › Figure 3/3D/MAX_IF_CD31_PdgfrbGFP_CLEC4F_CD68_portal_tract.jpg]

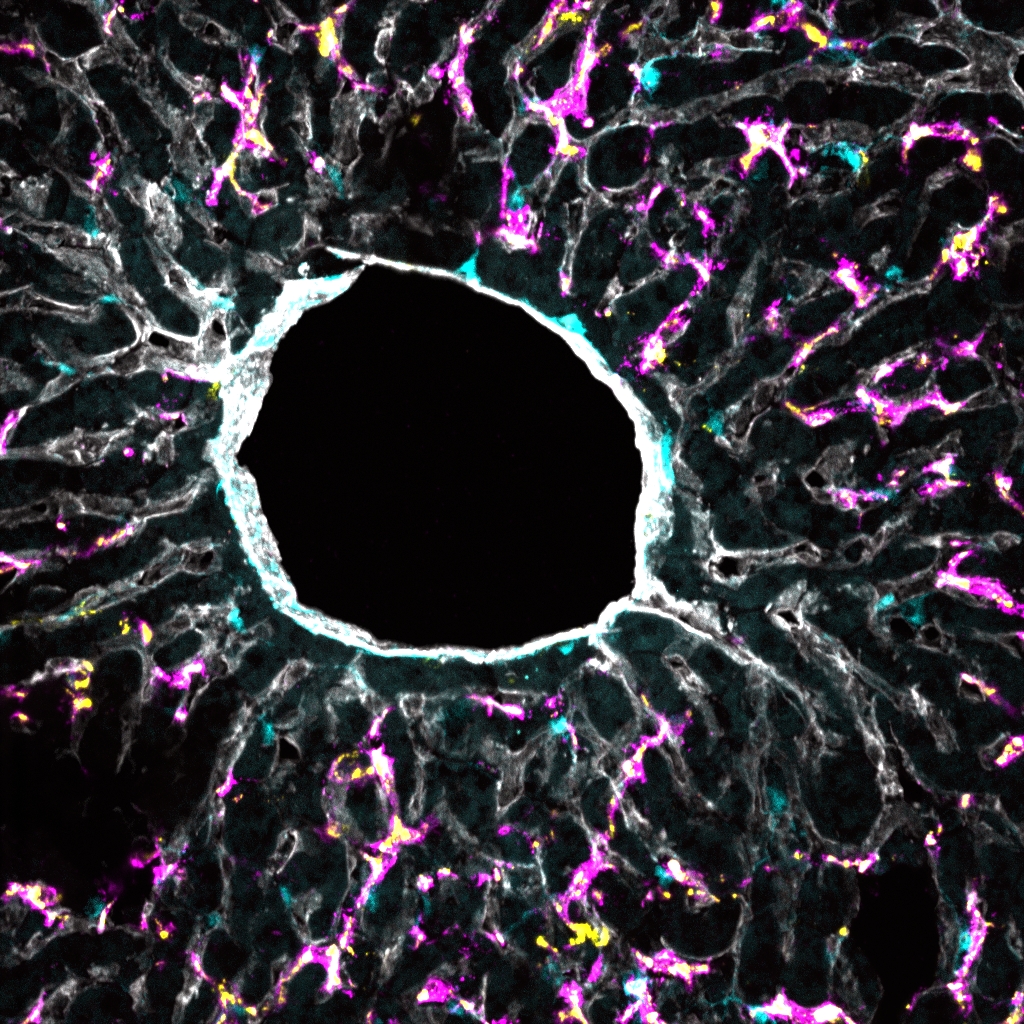

Supplement: Supplementary file 9 — Source data Fig. 3 [file 44319_2025_580_MOESM9_ESM.zip › Figure 3/3D/MAX_IF_CD31_PdgfrbGFP_CLEC4F_CD68_central_vein.jpg]

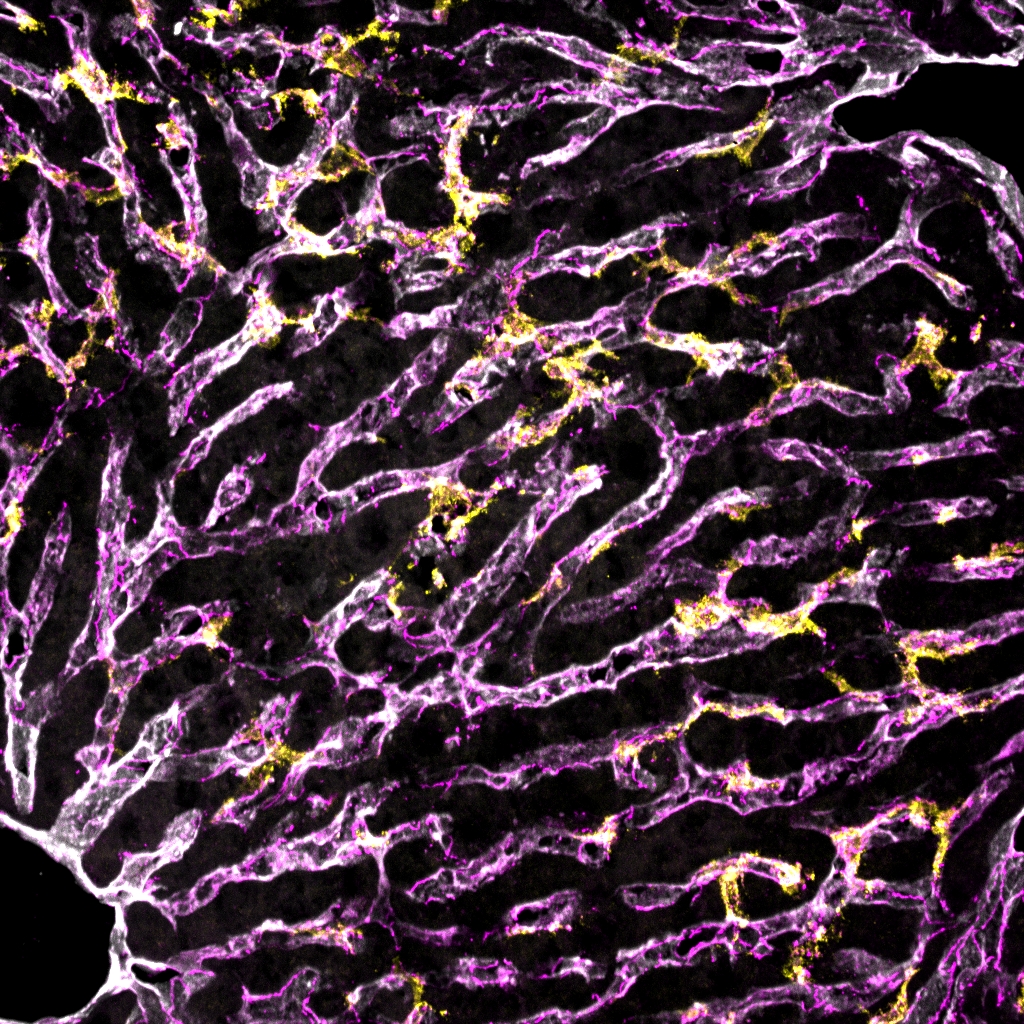

Supplement: Supplementary file 9 — Source data Fig. 3 [file 44319_2025_580_MOESM9_ESM.zip › Figure 3/3F/MAX_IF_CD31_CLEC4F_CDH5.jpg]

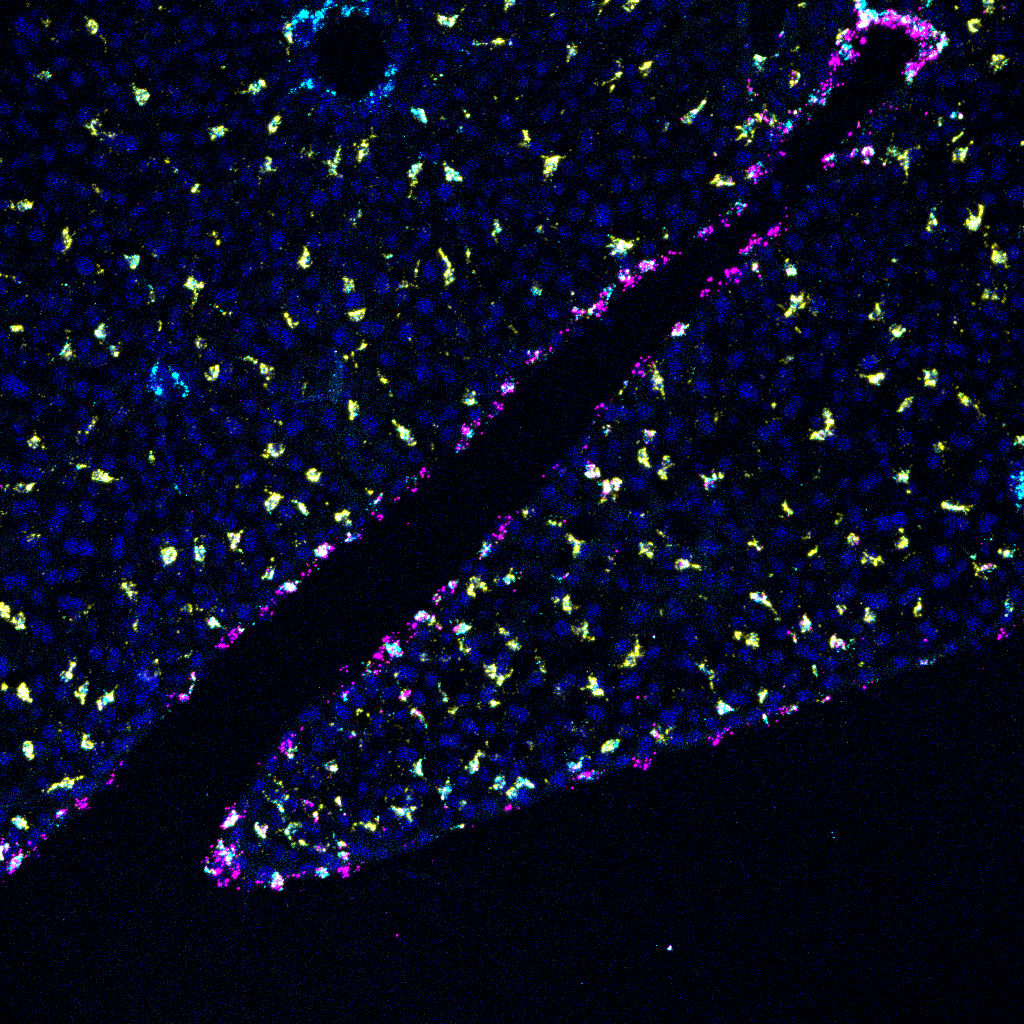

Supplement: Supplementary file 10 — Source data Fig. 4 [file 44319_2025_580_MOESM10_ESM.zip › Figure 4/4E/MAX_ISH_Nuclei_Pdgfrb_Wt1_Reln_liver_edge.tif]

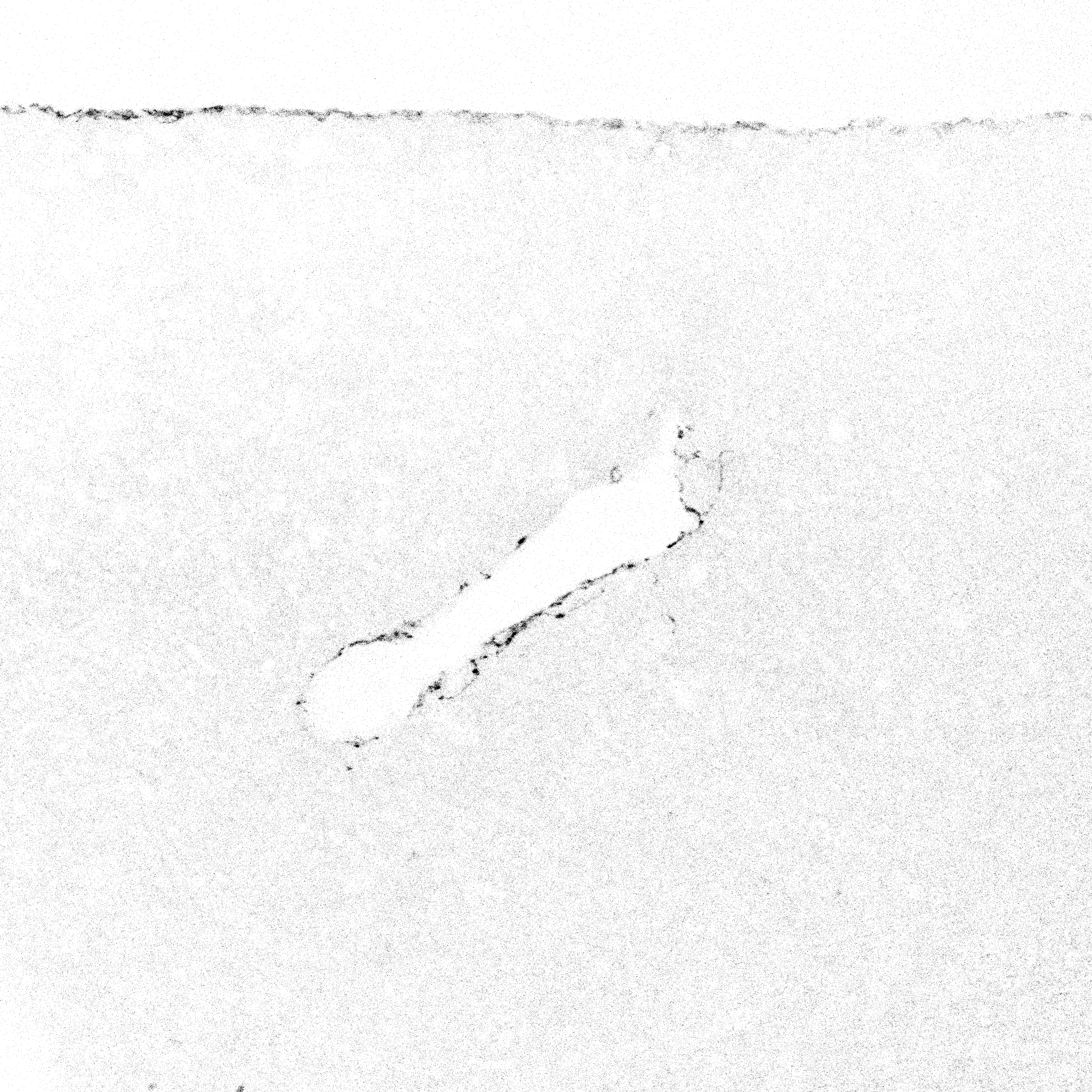

Supplement: Supplementary file 10 — Source data Fig. 4 [file 44319_2025_580_MOESM10_ESM.zip › Figure 4/4L/MAX_IF_NCAM1_liver_edge.jpg]

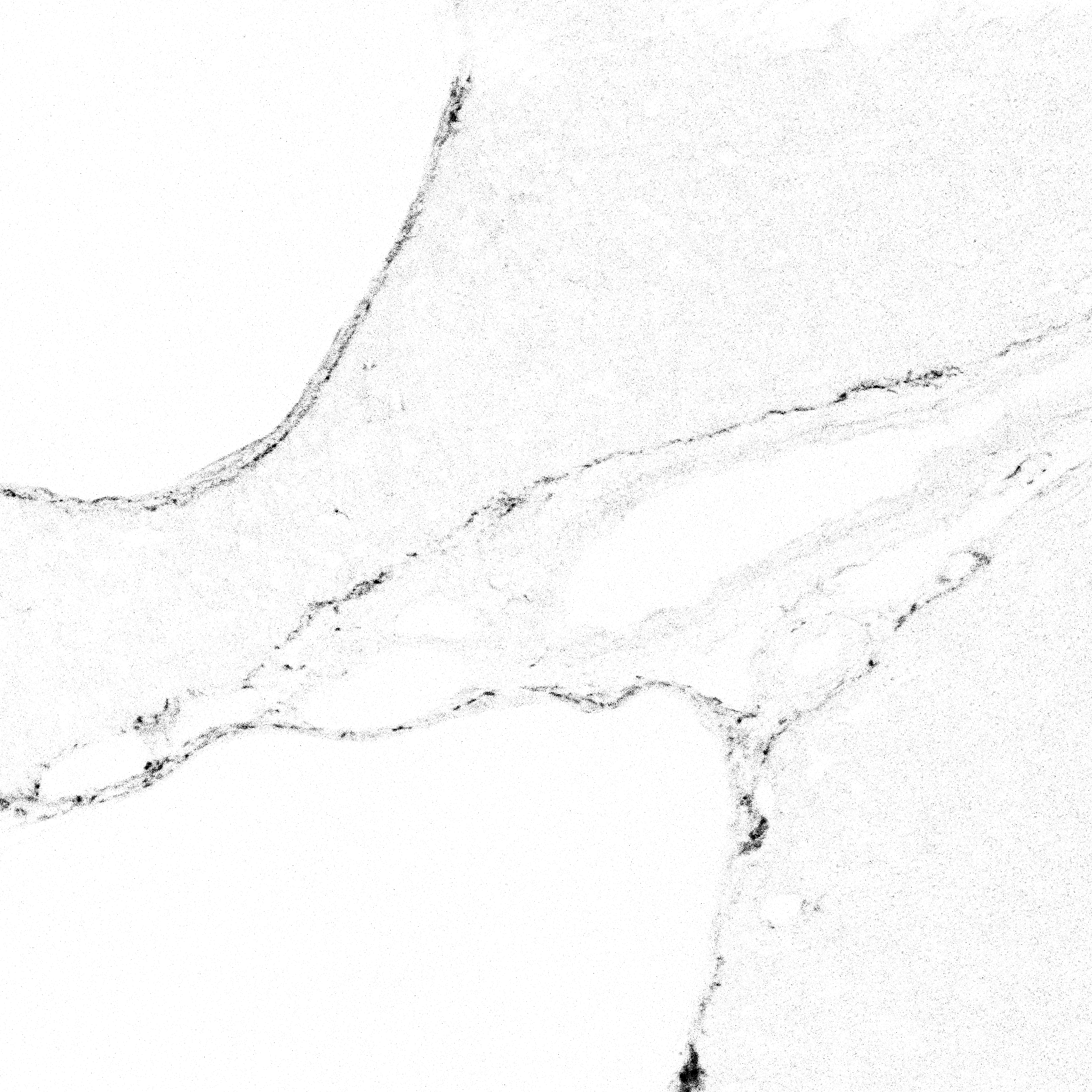

Supplement: Supplementary file 10 — Source data Fig. 4 [file 44319_2025_580_MOESM10_ESM.zip › Figure 4/4M/MAX_IF_NCAM1.jpg]

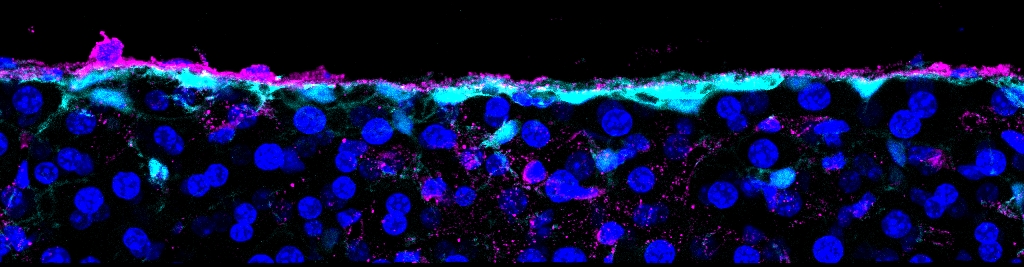

Supplement: Supplementary file 10 — Source data Fig. 4 [file 44319_2025_580_MOESM10_ESM.zip › Figure 4/4G/Angled_snap_shot_from_3D_rendering_IF_Nuclei_PdgfrbGFP_GPM6A_NCAM1.jpg]

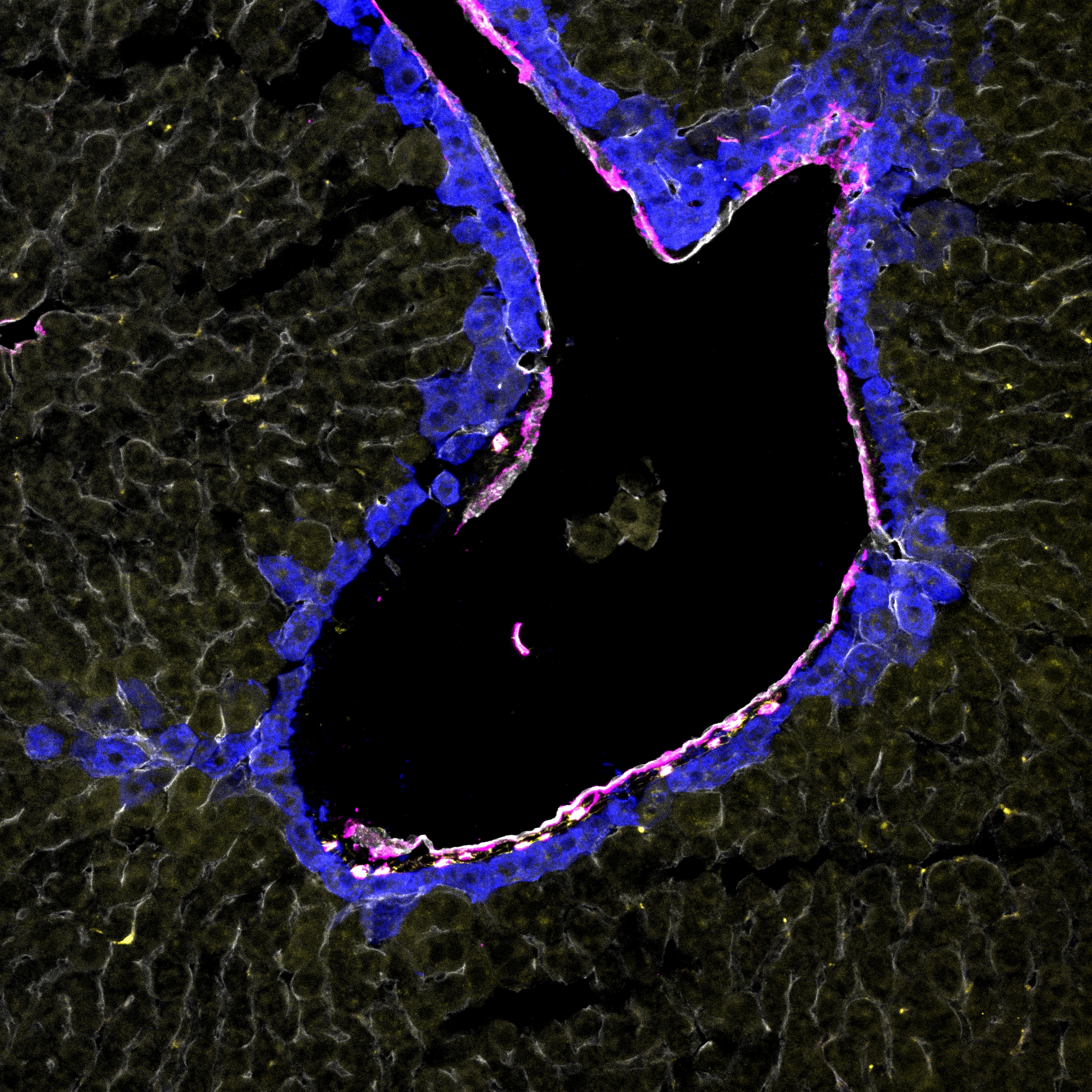

Supplement: Supplementary file 11 — Source data Fig. 5 [file 44319_2025_580_MOESM11_ESM.zip › Figure 5/5F/MAX_IF_aSMA_CD31_NPNT_GLUL_central_vein.jpg]

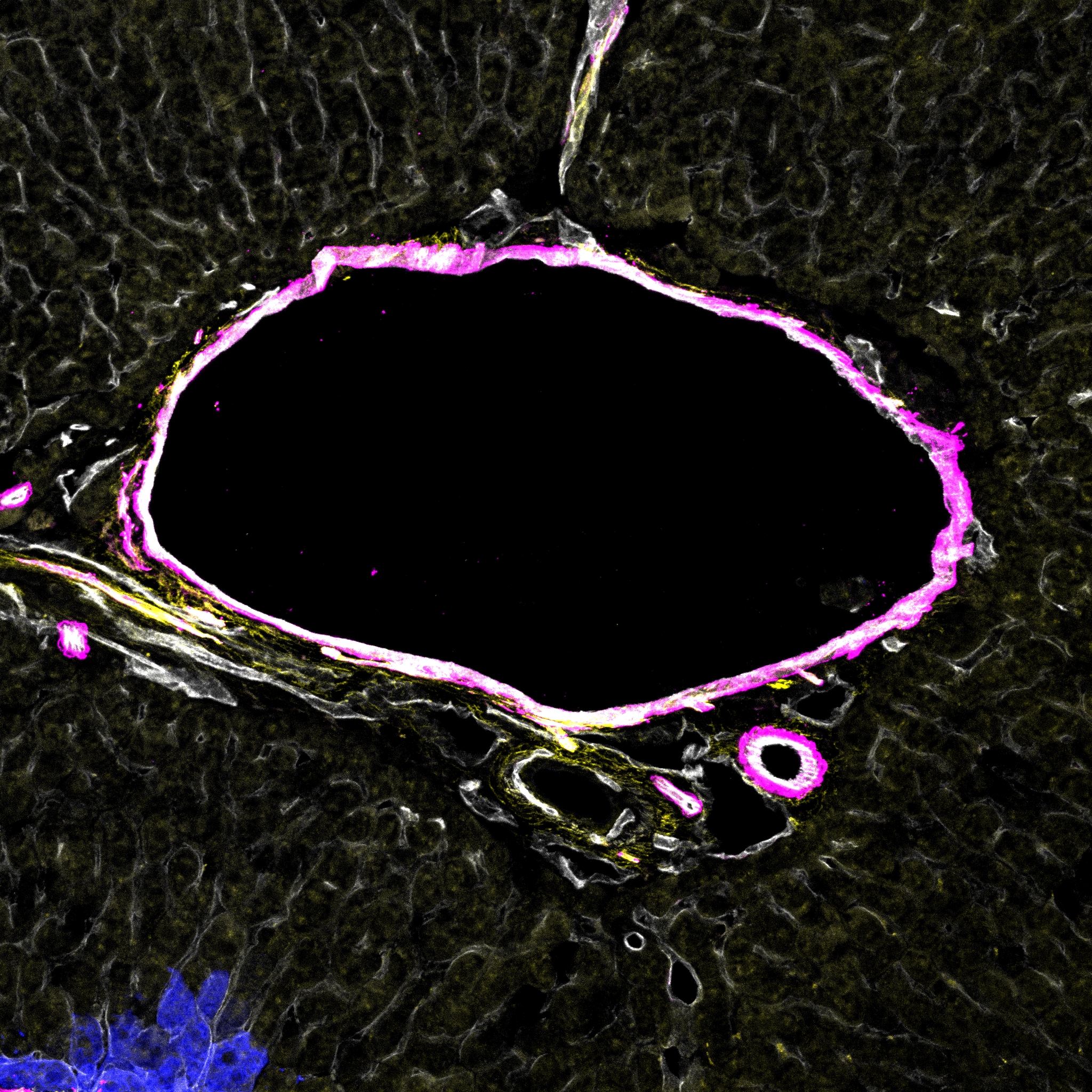

Supplement: Supplementary file 11 — Source data Fig. 5 [file 44319_2025_580_MOESM11_ESM.zip › Figure 5/5F/MAX_IF_aSMA_CD31_NPNT_GLUL_portal_tract.jpg]

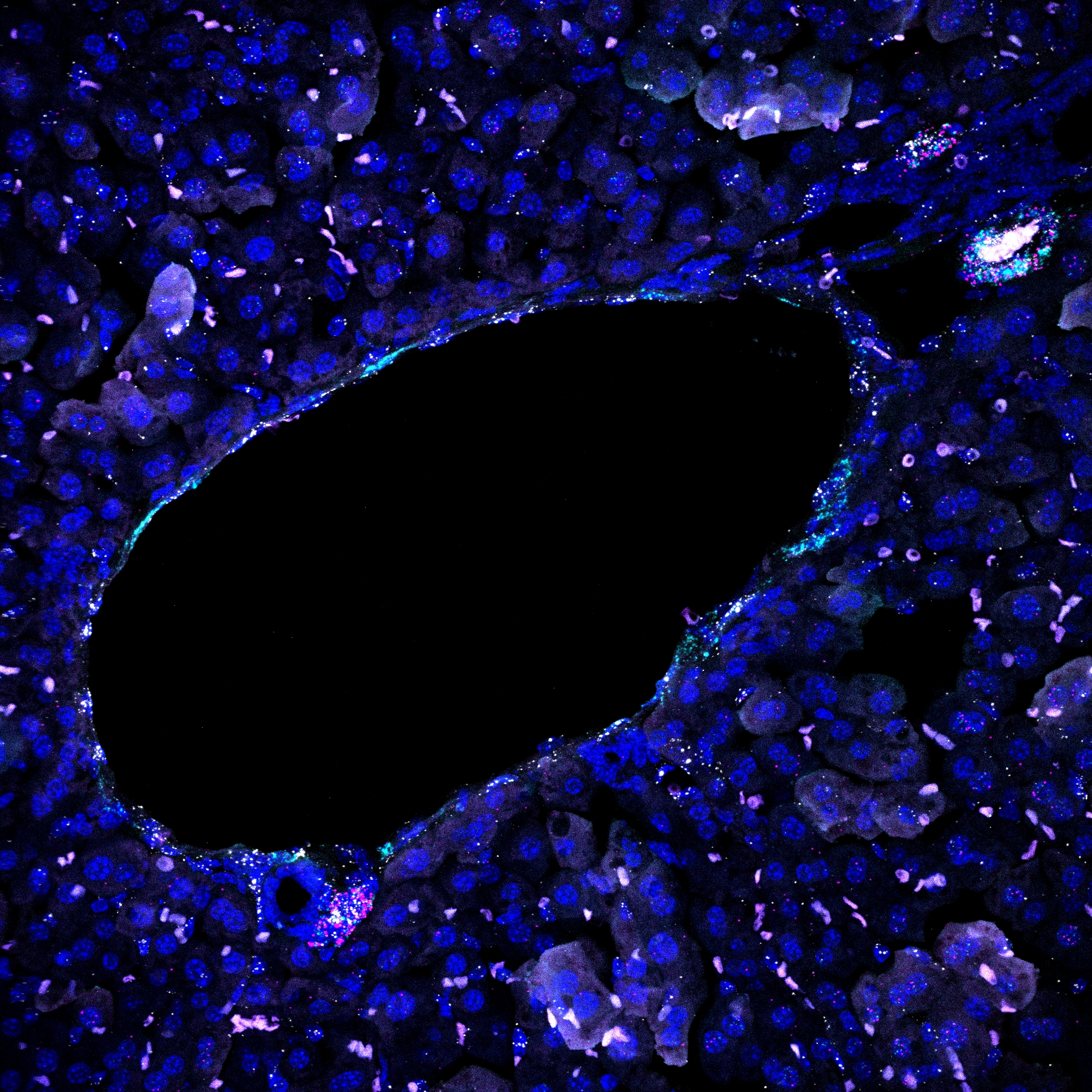

Supplement: Supplementary file 11 — Source data Fig. 5 [file 44319_2025_580_MOESM11_ESM.zip › Figure 5/5J/MAX_ISH_Nuclei_Acta2_Pln_Pecam1_portal_tract.jpg]

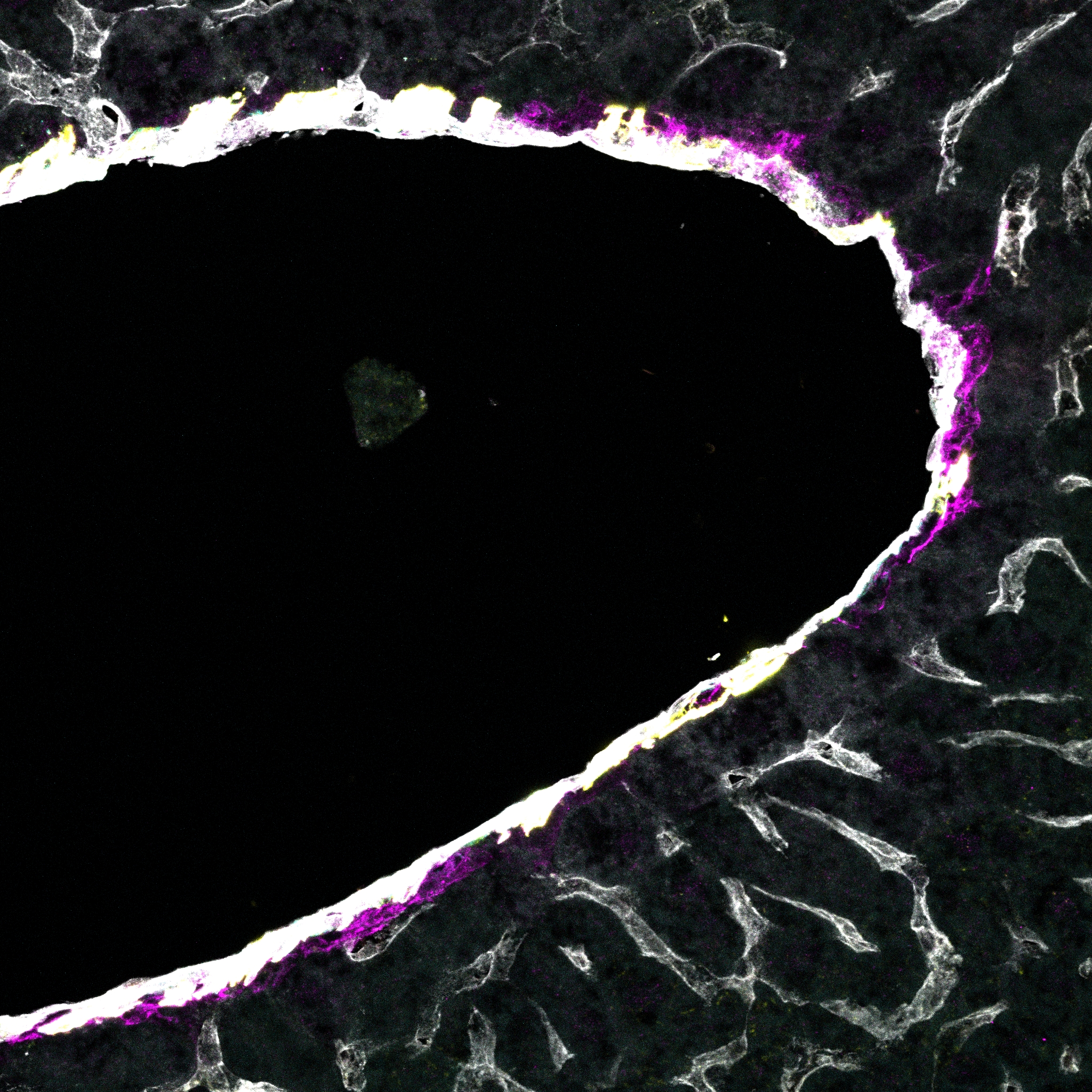

Supplement: Supplementary file 12 — Source data Fig. 6 [file 44319_2025_580_MOESM12_ESM.zip › Figure 6/6I/MAX_IF_aSMA_CD31_CNN1_NCAM1_central_vein.jpg]

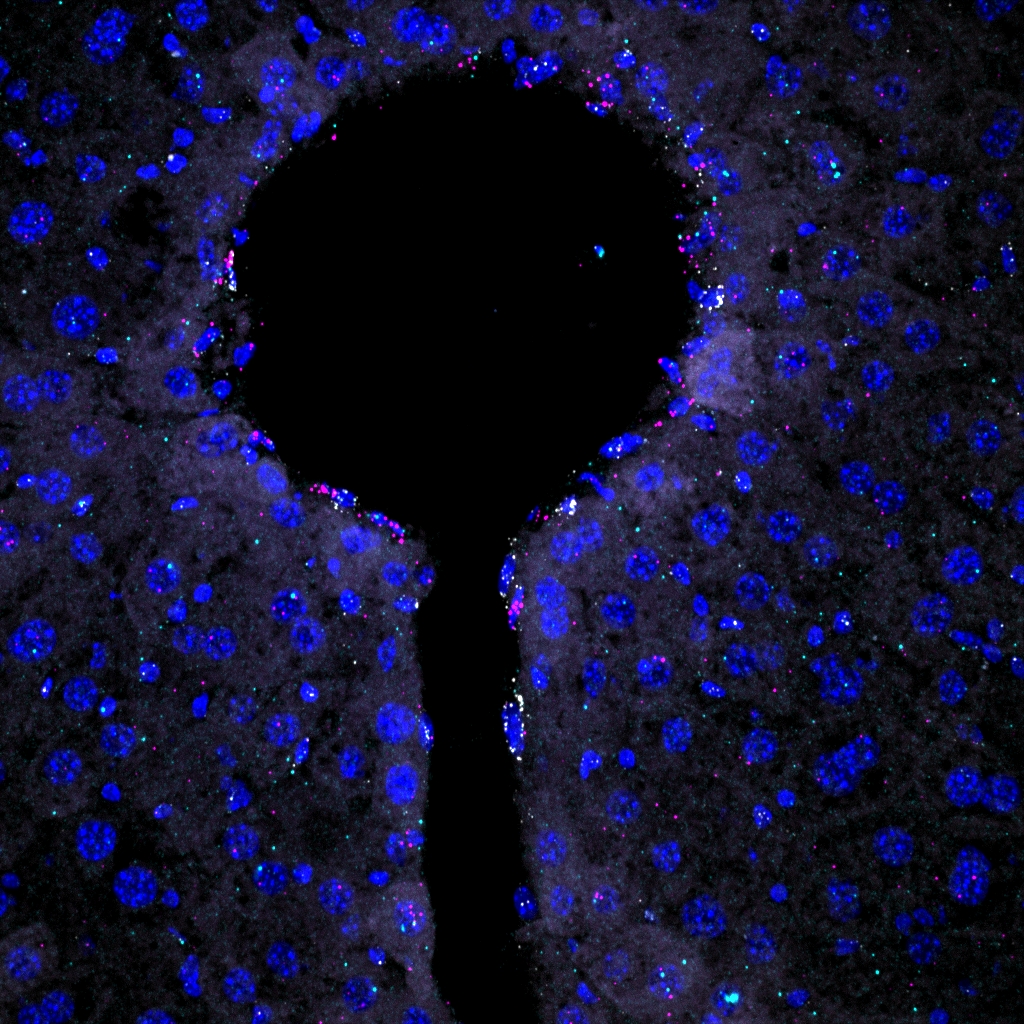

Supplement: Supplementary file 12 — Source data Fig. 6 [file 44319_2025_580_MOESM12_ESM.zip › Figure 6/6G/MAX_ISH_Nuclei_Acta2_Chrdl1_Pecam1_central_vein.jpg]

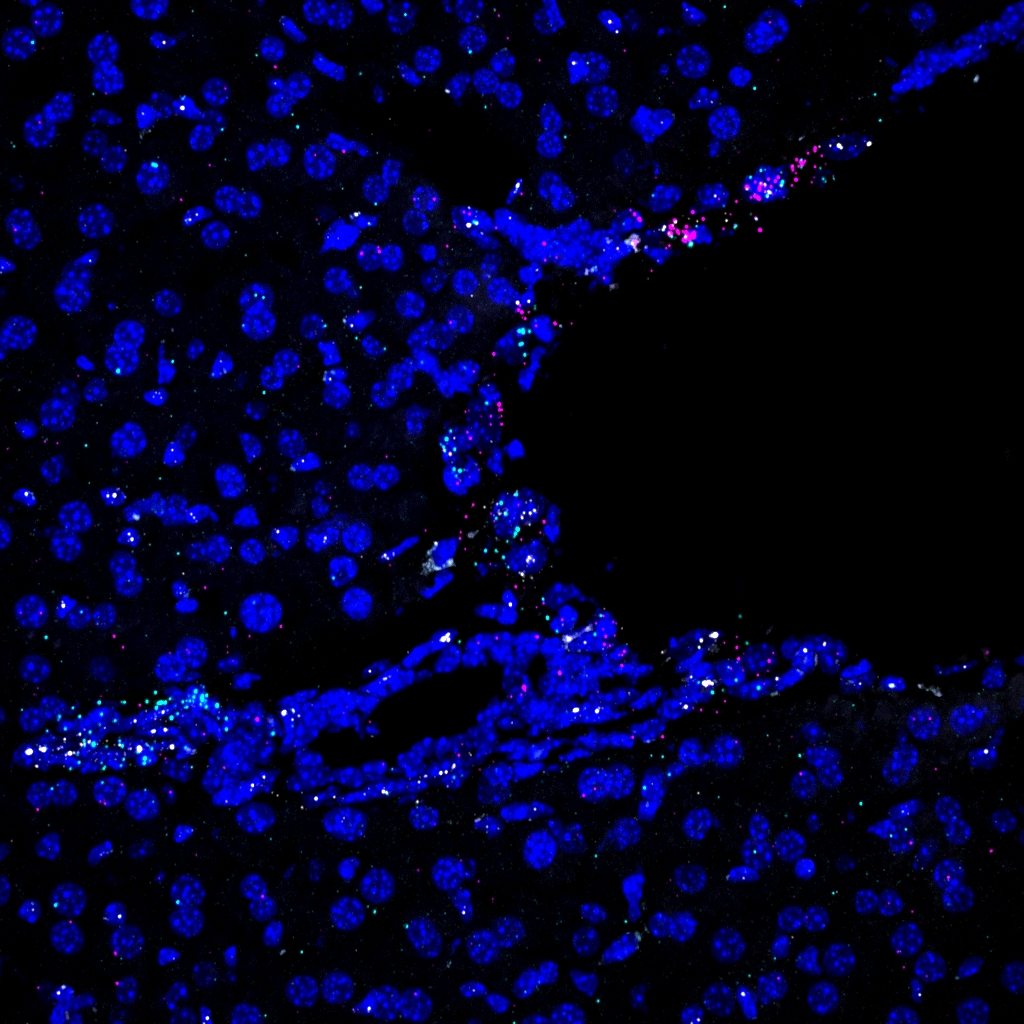

Supplement: Supplementary file 12 — Source data Fig. 6 [file 44319_2025_580_MOESM12_ESM.zip › Figure 6/6G/MAX_ISH_Nuclei_Acta2_Chrdl1_Pecam1_portal_tract.jpg]

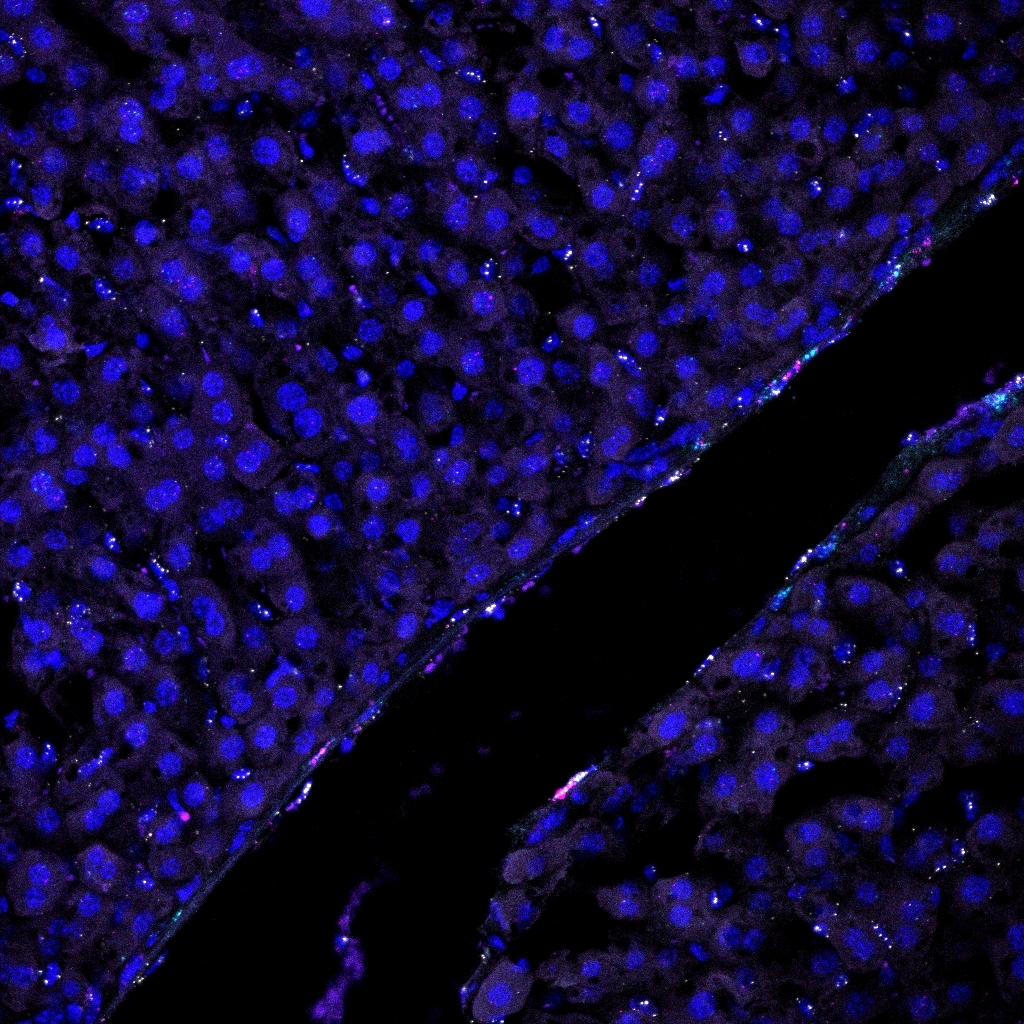

Supplement: Supplementary file 12 — Source data Fig. 6 [file 44319_2025_580_MOESM12_ESM.zip › Figure 6/6E/MAX_ISH_Nuclei_Acta2_Ccn3_Pecam1_central_vein.jpg]

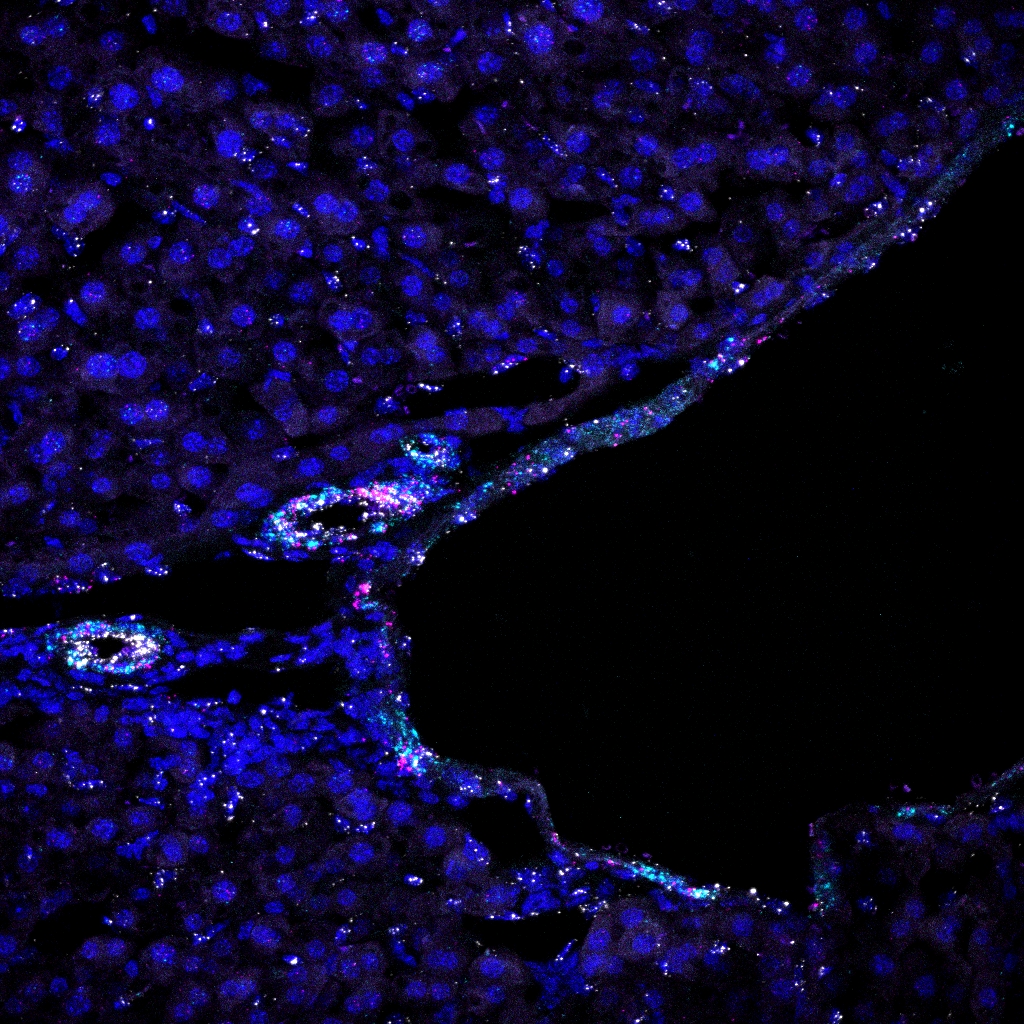

Supplement: Supplementary file 12 — Source data Fig. 6 [file 44319_2025_580_MOESM12_ESM.zip › Figure 6/6E/MAX_ISH_Nuclei_Acta2_Ccn3_Pecam1_portal_tract.jpg]

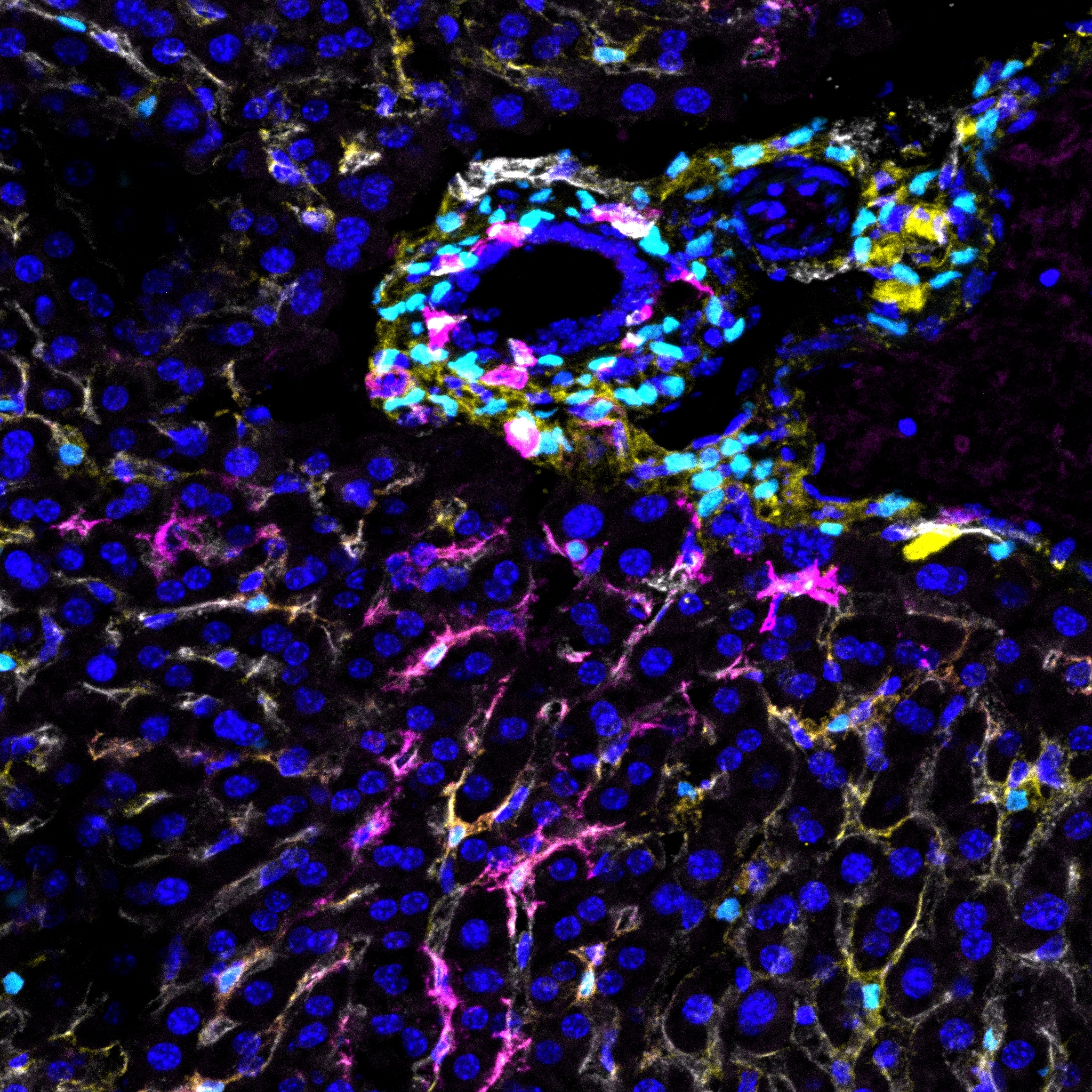

Supplement: Supplementary file 12 — Source data Fig. 6 [file 44319_2025_580_MOESM12_ESM.zip › Figure 6/6K/MAX_IF_Nuclei_PdgfraH2BGFP_ACE2_NGFR_portal_tract.jpg]

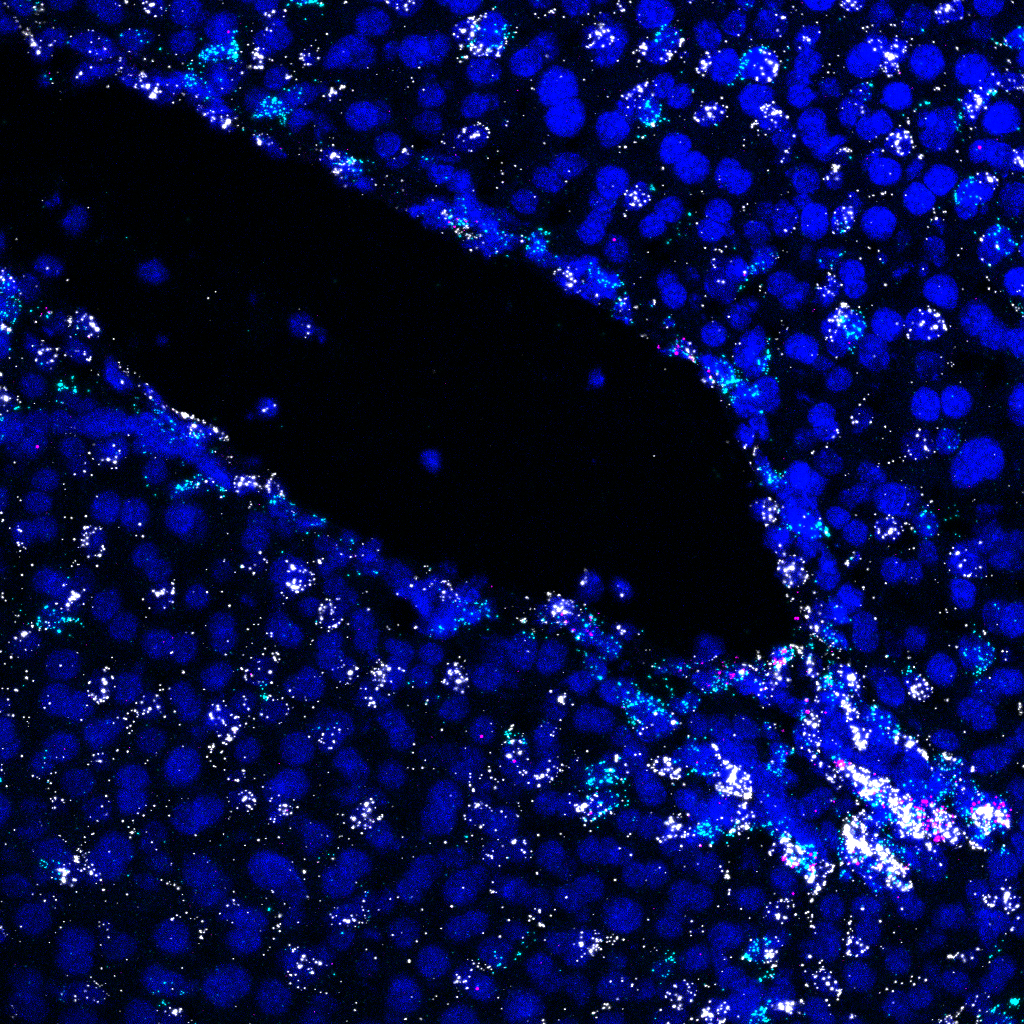

Supplement: Supplementary file 12 — Source data Fig. 6 [file 44319_2025_580_MOESM12_ESM.zip › Figure 6/6C/MAX_ISH_Nuclei_Pdgfrb_Ephx3_Pecam1_portal_tract.tif]

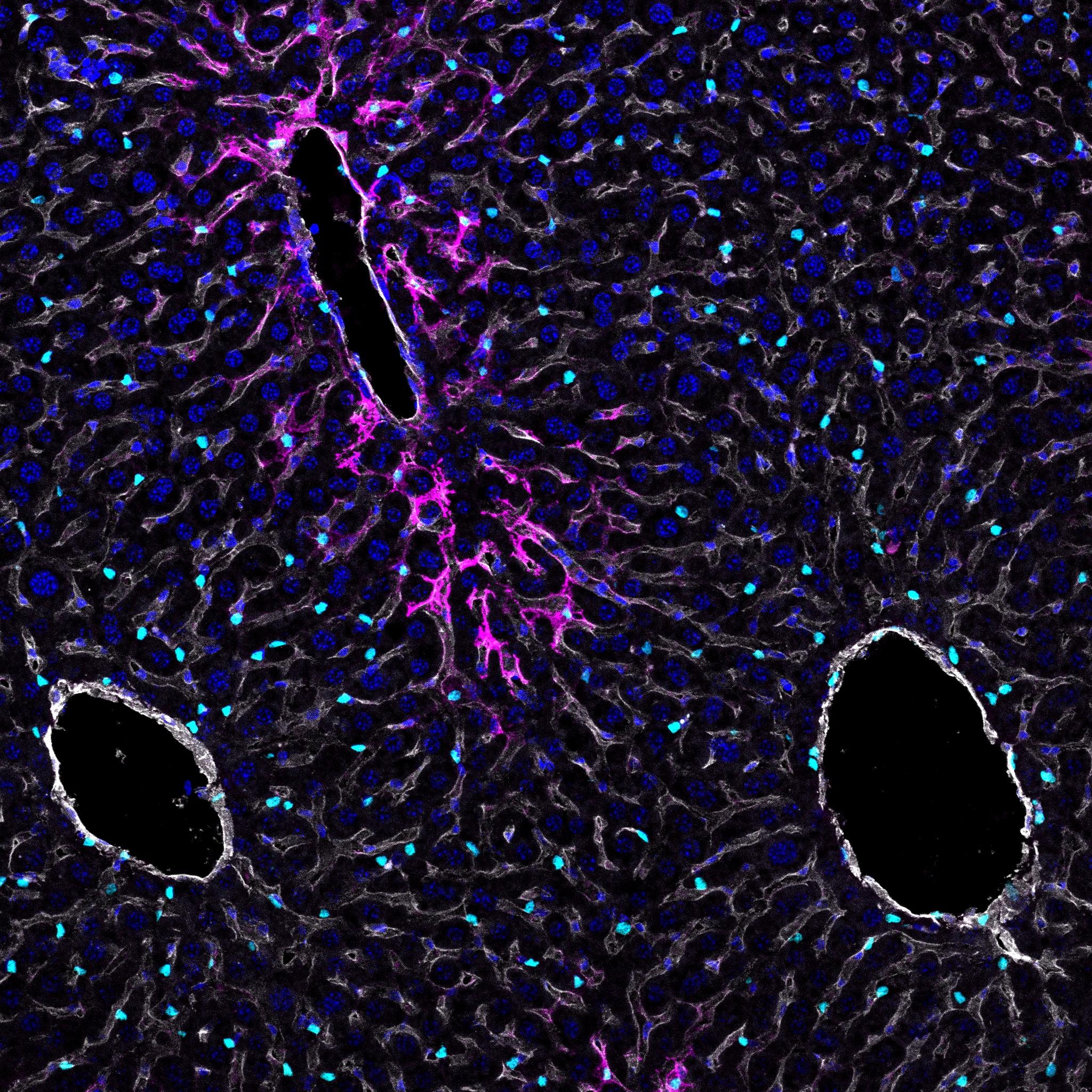

Supplement: Supplementary file 13 — Source data Fig. 7 [file 44319_2025_580_MOESM13_ESM.zip › Figure 7/7J/MAX_IF_Nuclei_PdgfraHebGFP_CD31_ACE2.jpg]

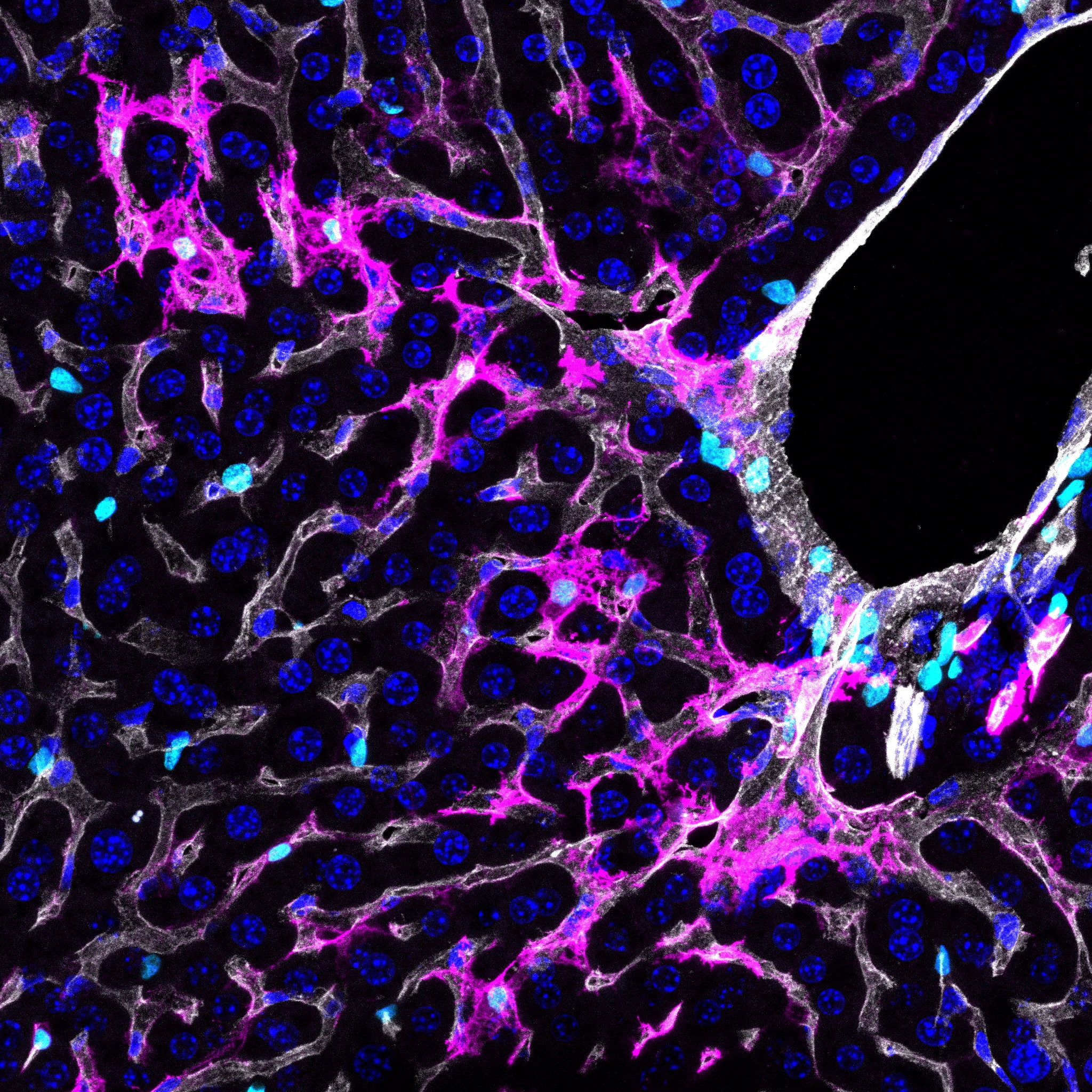

Supplement: Supplementary file 13 — Source data Fig. 7 [file 44319_2025_580_MOESM13_ESM.zip › Figure 7/7J/MAX_IF_Nuclei_PdgfraH2BGFP_CD31_ACE2_central_vein.jpg]

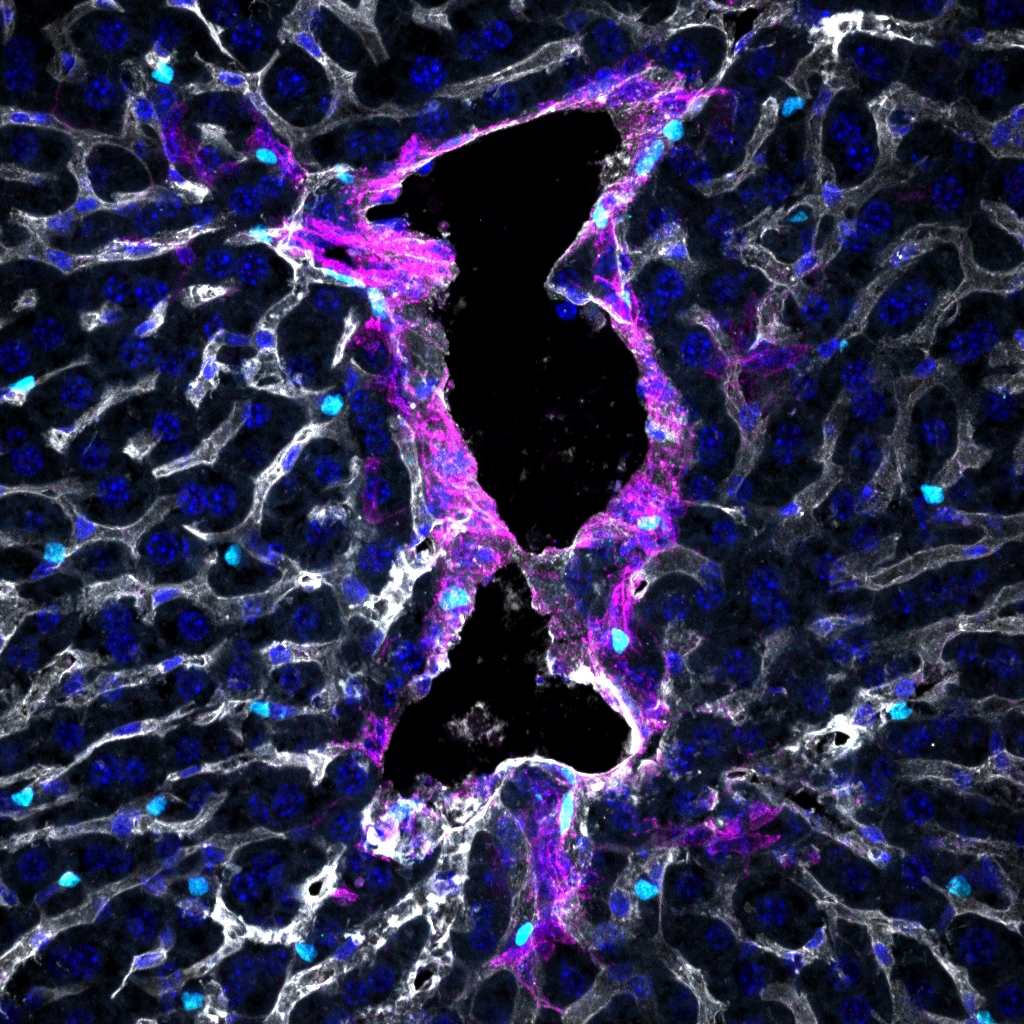

Supplement: Supplementary file 13 — Source data Fig. 7 [file 44319_2025_580_MOESM13_ESM.zip › Figure 7/7M/MAX_IF_Nuclei_PdgfraH2BGFP_EMCN_CDH3_portal_tract.jpg]

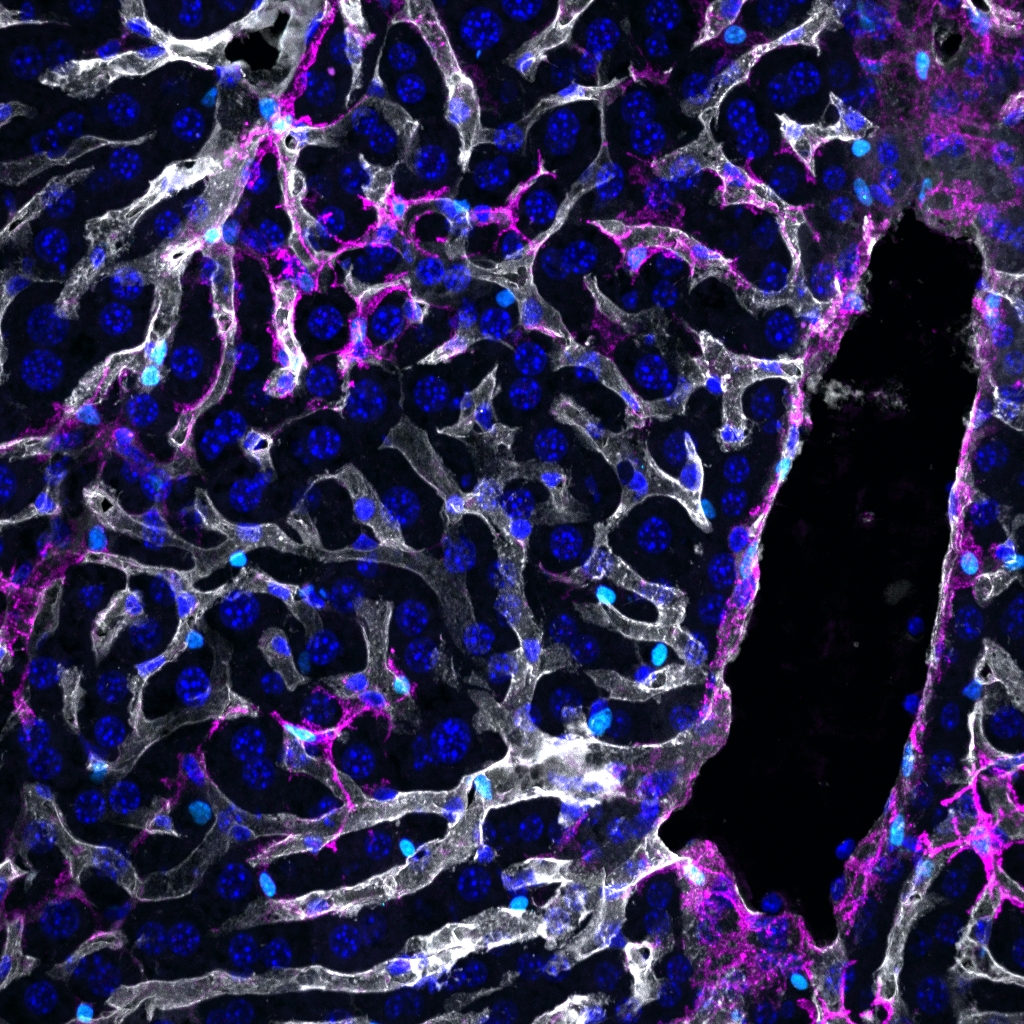

Supplement: Supplementary file 13 — Source data Fig. 7 [file 44319_2025_580_MOESM13_ESM.zip › Figure 7/7M/MAX_IF_Nuclei_PdgfraH2bGFP_EMCN_CDH3_central_vein.jpg]

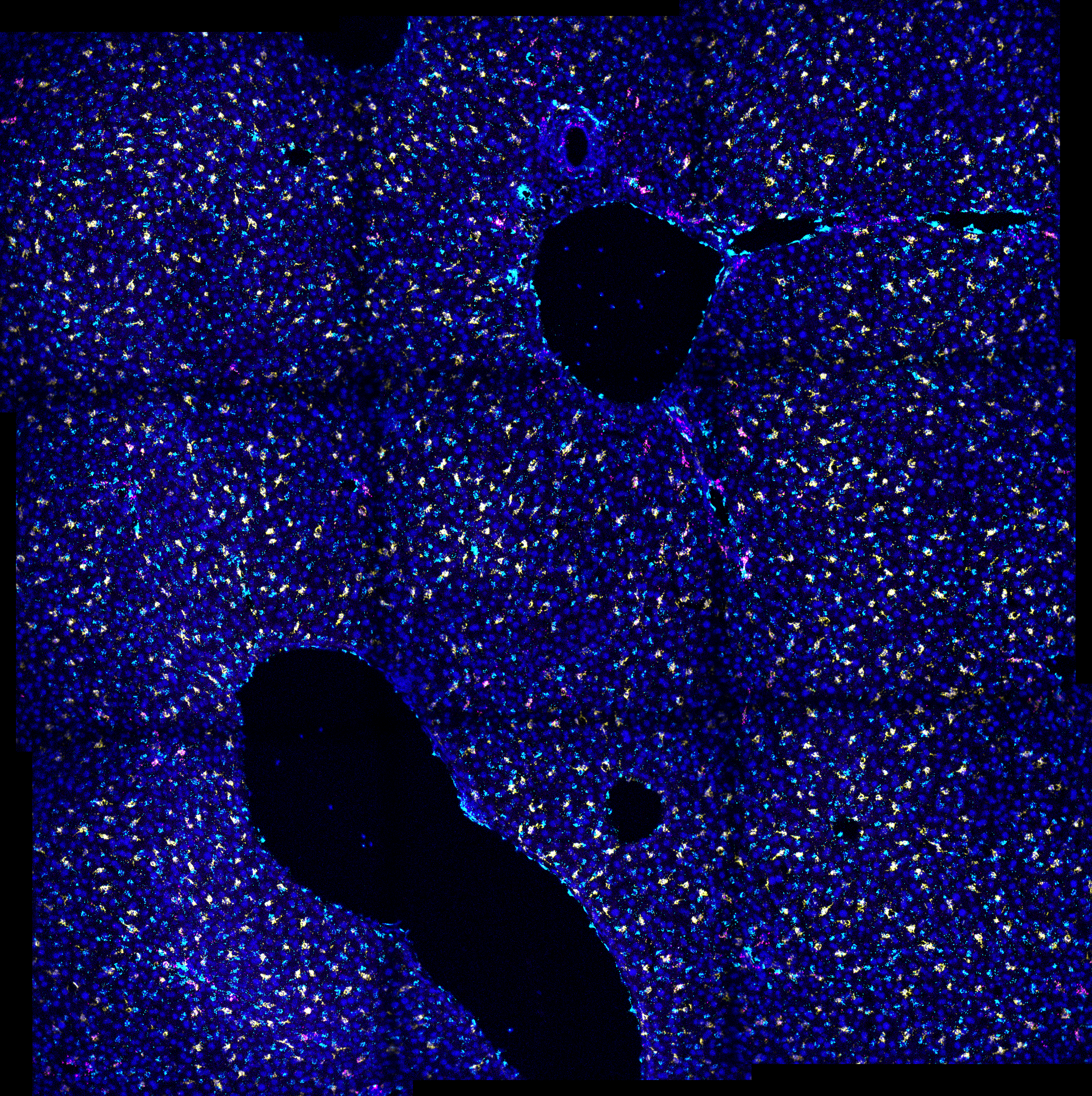

Supplement: Supplementary file 13 — Source data Fig. 7 [file 44319_2025_580_MOESM13_ESM.zip › Figure 7/7K/MAX_ISH_Nuclei_Pecam1_Ace2_Reln_tile.tif]

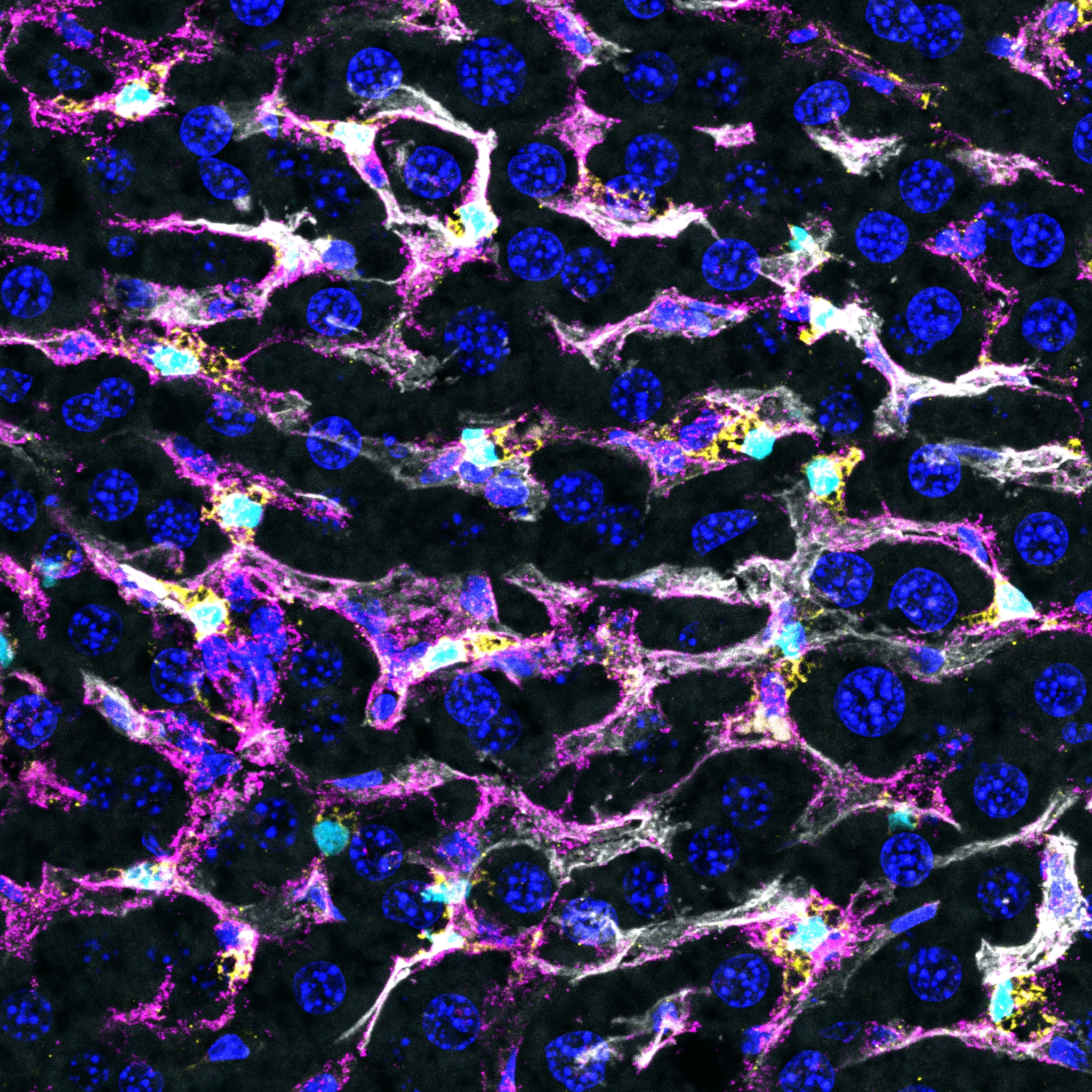

Supplement: Supplementary file 13 — Source data Fig. 7 [file 44319_2025_580_MOESM13_ESM.zip › Figure 7/7F/MAX_IF_Nuclei_LYVE1_PdgfraH2bGFP_RELN_PLVAP_boxed_area_from_Fig_EV5G.jpg]

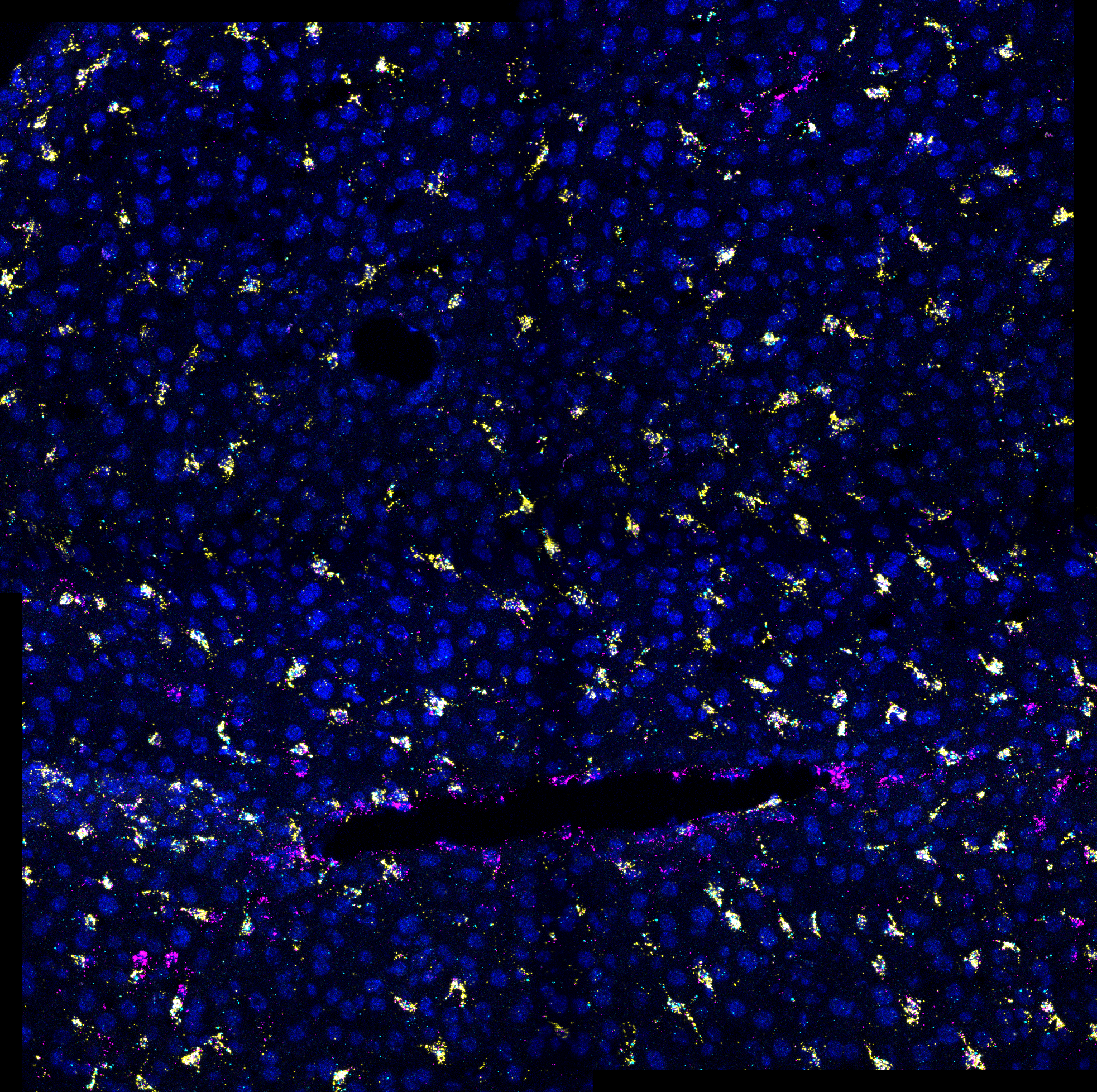

Supplement: Supplementary file 13 — Source data Fig. 7 [file 44319_2025_580_MOESM13_ESM.zip › Figure 7/7H/MAX_ISH_Nuclei_Hhip_Rspo3_Reln_tile.tif]
