# Supplementary material for: A comprehensive molecular atlas of the mesenchymal cell types in the mouse liver
Source: EMBO Rep. 2025 Sep 15;26(21):5326–59. doi: 10.1038/s44319-025-00580-9 (PMC12592516; doi:10.1038/s44319-025-00580-9)
Supplement: Supplementary file 14 — Expanded View Figures [file 44319_2025_580_MOESM14_ESM.pdf]

Expanded View Figures

Figure EV1. Cell type class identification and analysis of parenchymal cell dataset.

(**A**) UMAP visualization of the expression level of selected canonical marker genes representative for the cell type class identification. (**B**) UMAP visualization of the clustering result for the parenchymal cell (hepatocytes and cholangiocytes) datasets. (**C**, **D**) UMAP visualization of the expression level of exemplary genes identified as differentially expressed in the hepatocytes along the portal-central axis (**C**), or in a distinct hepatocyte subpopulation (**D**). (**E**) Heat map showing genes with enriched expression in cholangiocytes compared to hepatocytes. (**F**) Violin plots showing the expression levels of genes belonging to the GO term cell-cell junction organization (left panel) or steroid metabolic process (right panel), which are enriched in cholangiocytes when compared to the complete dataset (compare to Appendix Fig. S2B, n refers to cells: 7 = 205, 11 = 192, 1 = 415, 3 = 561, 9 = 394, 4 = 138, 6 = 119, 8 = 276, 12 = 199, 10 = 184, 5 = 279, 13 = 283, 14 = 116).

 © The Author(s)

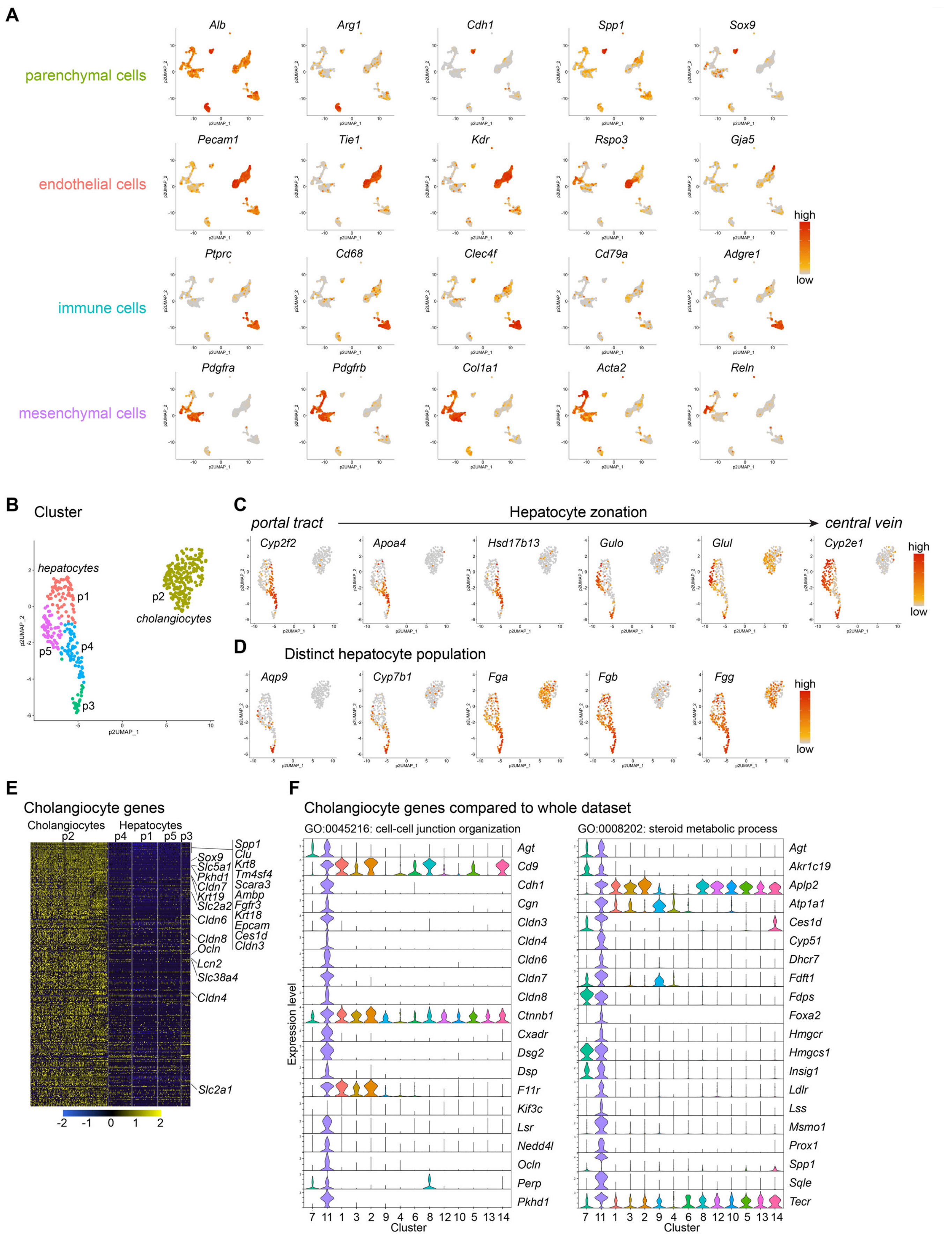
A
parenchymal cells
endothelial cells
immune cells
mesenchymal cells
Alb Arg1 Cdh1 Spp1 Sox9
Pecam1 Tie1 Kdr Rspo3 Gja5
Ptprc Cd68 Clec4f Cd79a Adgre1
Pdgfra Pdgfrb Col1a1 Acta2 Reln
high
low
B Cluster
hepatocytes
p1 p2 p3 p4 p5
cholangiocytes
C
portal tract
Hepatocyte zonation
central vein
Cyp2f2 Apoa4 Hsd17b13 Gulo Glul Cyp2e1
D
Distinct hepatocyte population
Aqp9 Cyp7b1 Fga Fgb Fgg
E
Cholangiocyte genes
Cholangiocytes Hepatocytes
p2 p4 p1 p5 p3
Spp1 Clu Sox9 Slc5a1 Krt8 Pkhd1 Tm4sf4 Cldn7 Scara3 Krt19 Ambp Slc2a2 Fgfr3 Cldn6 Krt18 Epcam Cldn8 Ces1d Ocln Cldn3 Lcn2 Slc38a4 Cldn4 Slc2a1
-2 -1 0 1 2
F
Cholangiocyte genes compared to whole dataset
GO:0045216: cell-cell junction organization
GO:0008202: steroid metabolic process
Expression level
Agt Cd9 Cdh1 Cgn Cldn3 Cldn4 Cldn6 Cldn7 Cldn8 Ctnnb1 Cxadr Dsg2 Dsp F11r Kif3c Lsr Nedd4l Ocln Perp Pkhd1
Agt Akr1c19 Aplp2 Atp1a1 Ces1d Cyp51 Dhcr7 Fdft1 Fdps Foxa2 Hmgcr Hmgcs1 Insig1 Ldlr Lss Msmo1 Prox1 Spp1 Sqle Tecr
7 11 1 3 2 9 4 6 8 12 10 5 13 14
Cluster

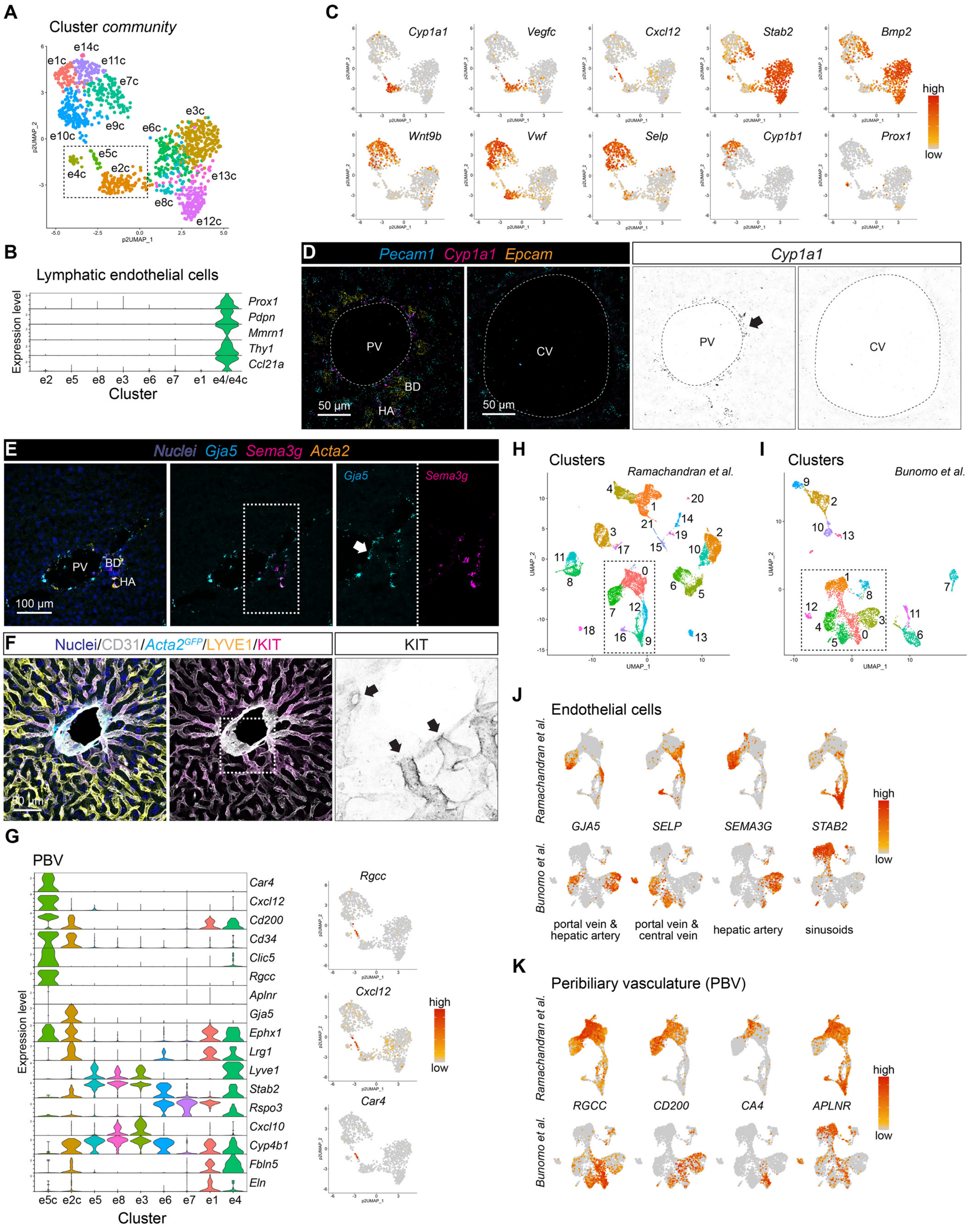
A
Cluster community
e1c
e14c
e11c
e7c
e9c
e10c
e3c
e6c
e5c
e4c
e2c
e13c
e8c
e12c
B
Lymphatic endothelial cells
Expression level
Prox1
Pdpn
Mmrn1
Thy1
Ccl21a
e2 e5 e8 e3 e6 e7 e1 e4/e4c
Cluster
C
Cyp1a1
Vegfc
Cxcl12
Stab2
Bmp2
Wnt9b
Vwf
Selp
Cyp1b1
Prox1
high
low
D
Pecam1 Cyp1a1 Epcam
Cyp1a1
PV
CV
BD
HA
50 µm
E
Nuclei Gja5 Sema3g Acta2
Gja5
Sema3g
PV
BD
HA
100 µm
F
Nuclei/CD31/Acta2GFP/LYVE1/KIT
KIT
G
PBV
Car4
Cxcl12
Cd200
Cd34
Clic5
Rgcc
Aplnr
Gja5
Ephx1
Lrg1
Lyve1
Stab2
Rspo3
Cxcl10
Cyp4b1
Fbln5
Eln
Expression level
e5c e2c e5 e8 e3 e6 e7 e1 e4
Cluster
Rgcc
Cxcl12
Car4
high
low
H
Clusters
Ramachandran et al.
I
Clusters
Bunomo et al.
J
Endothelial cells
Ramachandran et al.
Bunomo et al.
GJA5
SELP
SEMA3G
STAB2
portal vein &
hepatic artery
portal vein &
central vein
hepatic artery
sinusoids
high
low
K
Peribiliary vasculature (PBV)
Ramachandran et al.
Bunomo et al.
RGCC
CD200
CA4
APLNR
high
low

Figure EV2. Analysis of endothelial cell subclusters and their relation to human liver single-cell data.

(**A**) UMAP visualization of the clustering results of the endothelial cell dataset using the pagoda2 community setting for high resolution clustering. (**B**) Violin plot showing genes expressed in lymphatic endothelial cells (cluster #e4/e4c, n refers to cells: e2 = 128, e5 = 151, e8 = 155, e3 = 236, e6 = 112, e7 = 150, e4/e4c = 22). (**C**) UMAP visualization of the expression levels of exemplary genes with zonated expression along the portal-central axis in hepatic endothelial cells, and *Prox1* as lymphatic endothelial cell marker. (**D**) ISH for *Pecam1*, *Cyp1a1*, and *Epcam* on a liver tissue section. Arrow indicates *Cyp1a1*-positive portal vein endothelial cells. (**E**) ISH for *Gja5*, *Sema3g*, and *Acta2* on a liver tissue section. Arrow highlights *Gja5* positive, *Sema3g* negative portal vein endothelial cells. (**F**) IF for CD31, LYVE1, and KIT on a liver tissue section from an *Acta2*^{GFP} reporter mouse. Arrows highlight KIT-positive last sinusoidal endothelial cells surrounding the central vein. (**G**) Violin plot showing the expression level of exemplary genes expressed by endothelial cells of the peribiliary vasculature (PBV) compared to other endothelial cell clusters (left panel, n refers to cells: e5c = 14, e2c = 114, e5 = 151, e8 = 155, e3 = 236, e6 = 112, e7 = 150, e1 = 152, e4 = 22). UMAP visualization of the expression level of peribiliary vasculature marker genes, *Rgcc*, *Cxcl12*, and *Car4* (right panel). (**H**, **I**) UMAP visualization of clustering results from human liver single-cell datasets GSE136103 (**H**) and GSE168933 (**I**). Cell clouds representing endothelial cells are indicated by the boxed area. (**J**, **K**) UMAP visualization of the magnified part of the UMAP landscapes from the human datasets showing the expression level of endothelial cell subtype markers: (**J**) *GJA5* for portal vein and hepatic artery, *SELP* for portal vein and central vein, *SEMA3G* for hepatic artery, and *STAB2* for sinusoids, or (**K**) *RGCC*, *CD200*, *CA4*, and *APLNR* for the peribiliary vasculature (PBV). PV portal vein, CV central vein, HA hepatic artery, BD bile duct. Scale bars are indicated in the respective image panels.

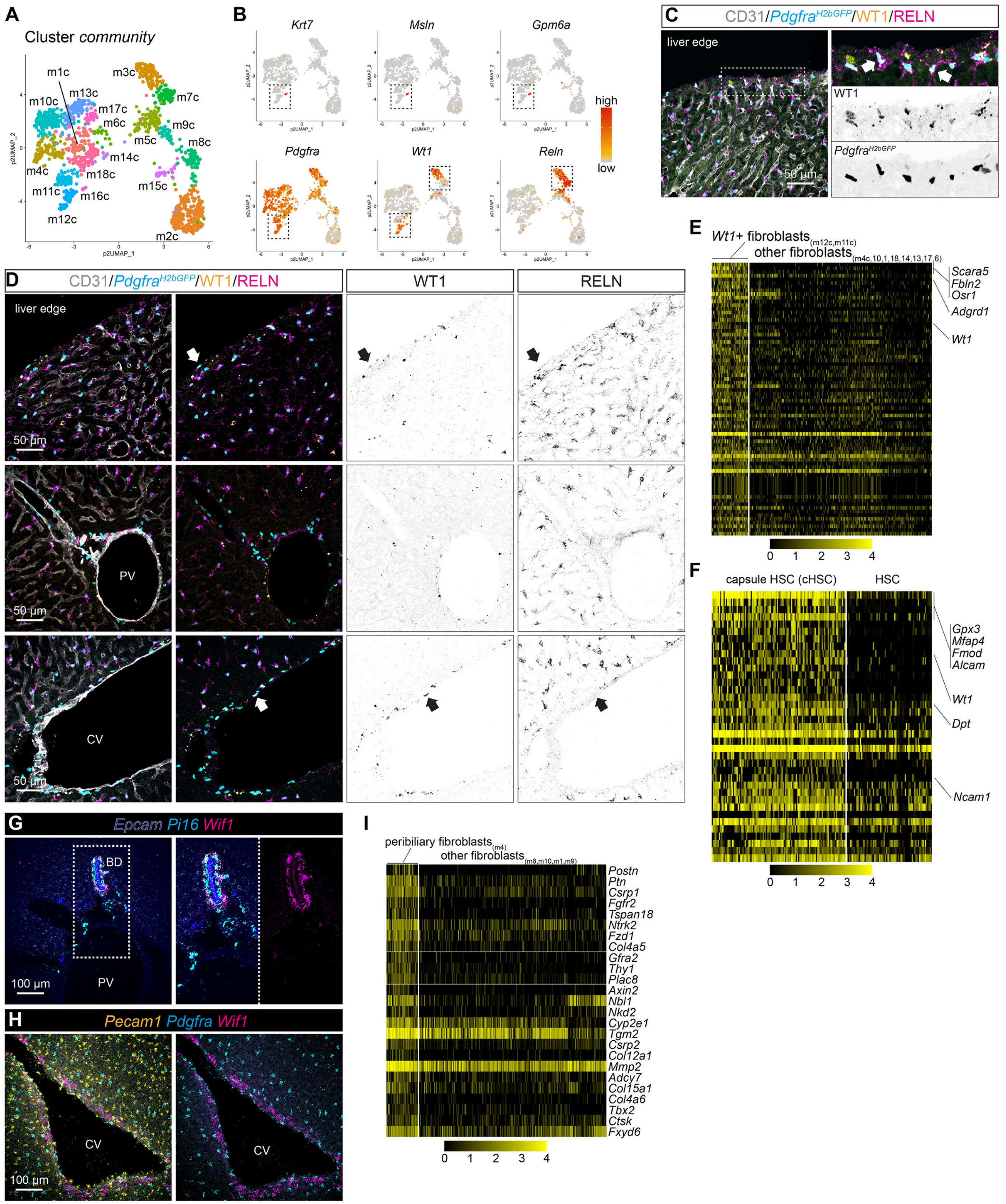
A
Cluster community
m1c
m3c
m10c
m13c
m7c
m17c
m6c
m9c
m5c
m8c
m14c
m4c
m18c
m11c
m16c
m15c
m12c
m2c
p2UMAP_1
p2UMAP_2
B
Krt7
Msln
Gpm6a
Pdgfra
Wt1
Reln
high
low
C
CD31/Pdgfra H2bGFP/WT1/RELN
liver edge
WT1
Pdgfra H2bGFP
50 µm
D
CD31/Pdgfra H2bGFP/WT1/RELN
WT1
RELN
liver edge
50 µm
PV
CV
E
Wt1+ fibroblasts (m12c,m11c)
other fibroblasts (m4c,10,1,18,14,13,17,6)
Scara5
Fbln2
Osr1
Adgrd1
Wt1
0 1 2 3 4
F
capsule HSC (cHSC)
HSC
Gpx3
Mfap4
Fmod
Alcam
Wt1
Dpt
Ncam1
0 1 2 3 4
G
Epcam Pi16 Wif1
BD
100 µm
PV
H
Pecam1 Pdgfra Wif1
CV
100 µm
I
peribiliary fibroblasts (m4)
other fibroblasts (m8,m10,m1,m9)
Postn
Ptn
Csrp1
Fgfr2
Tspan18
Ntrk2
Fzd1
Col4a5
Gfra2
Thy1
Plac8
Axin2
Nbl1
Nkd2
Cyp2e1
Tgm2
Csrp2
Col12a1
Mmp2
Adcy7
Col15a1
Col4a6
Tbx2
Ctsk
Fxyd6
0 1 2 3 4

 © The Author(s)

Figure EV3. Marker genes and tissue validation for mesenchymal cell subpopulations; cHSC and peribiliary fibroblasts.

(**A**) UMAP visualization of the pagoda2 clustering result of the mesenchymal cell subset using the community setting for high resolution clustering. (**B**) UMAP visualization of the expression level of selected genes within the indicated boxed areas of clusters #m3 (m3c, m7c) and #m9 (m11c, m12c, m16c). (**C**) IF for CD31, WT1, and RELN on liver tissue section from a *Pdgfra*^{H2bGFP} reporter mouse. The arrows indicate WT1, RELN, GFP triple-positive cells. (**D**) IF for CD31, WT1, and RELN on a liver tissue sections from a *Pdgfra*^{H2bGFP} reporter mouse focusing on the liver edge (upper panel), the portal tract (middle panel), or the central vein (lower panel). Arrows highlight WT1 *Pdgfra*^{H2bGFP} double-positive cells. (**E**) Heat map showing the expression of genes that exhibit enriched expression in *Wt1*+ fibroblasts (clusters #m11c, m12c), compared to all other fibroblast populations. (**F**) Heat map showing the expression of genes that exhibit enriched expression in cHSC (cluster #m3c), compared to HSC (cluster #m7c). (**G**) ISH for *Epcam*, *Pi16*, and *Wif1* on a liver tissue section. (**H**) ISH for *Pecam1*, *Pdgfra*, and *Wif1* on a liver tissue section. (**I**) Heat map showing the genes that exhibit enriched expression in peribiliary fibroblasts (cluster #m4), compared to all other fibroblast populations. PV portal vein, BD bile duct, HA hepatic artery, CV central vein. Scale bars are indicated in the respective image panels.

© The Author(s)

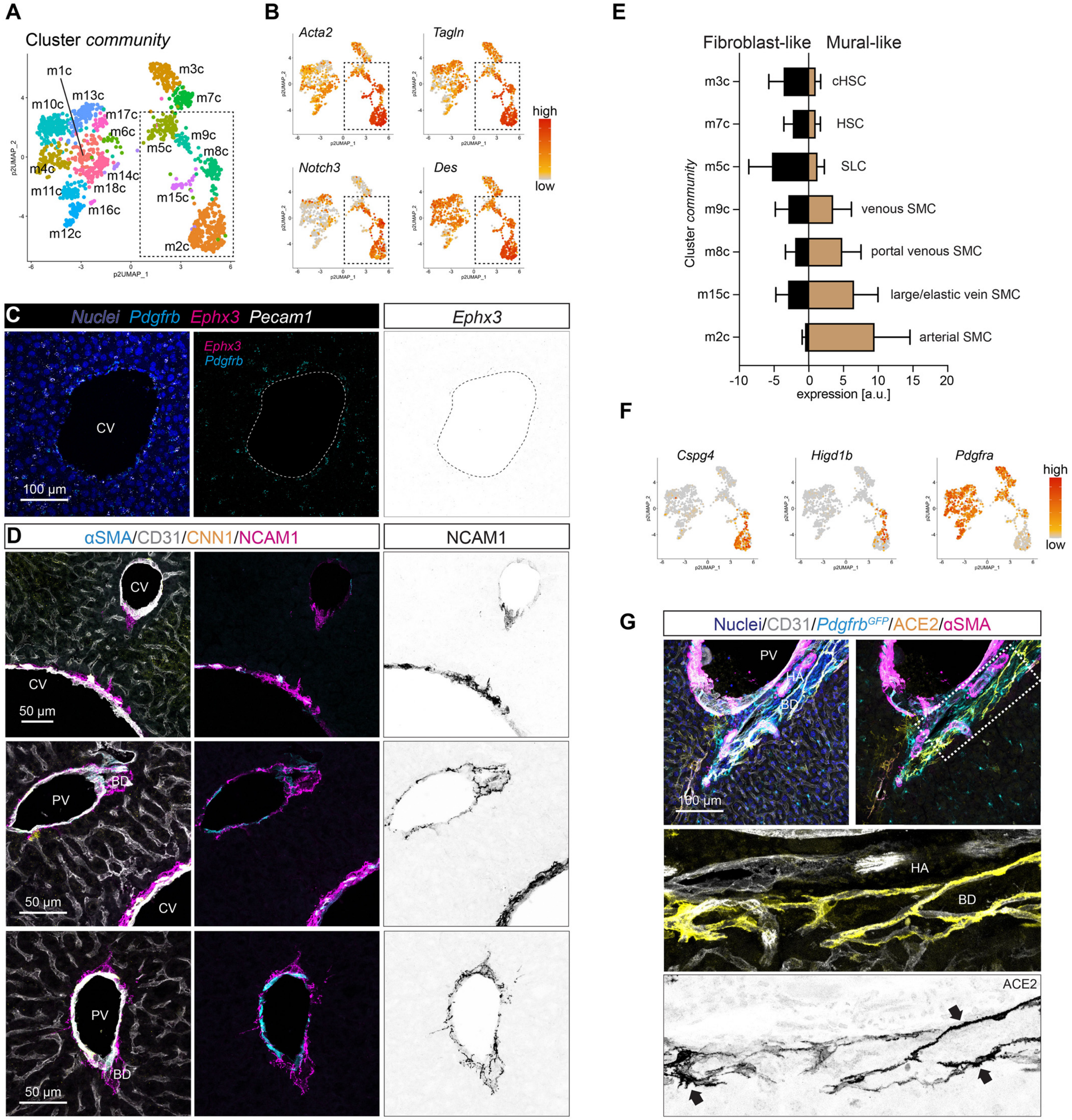

Figure EV4. Analysis of mural cell subpopulations.

(**A**) UMAP visualization of the clustering result of the mesenchymal cell dataset using the community setting for high resolution clustering, with the cell clouds containing vascular mural cell types indicated in the boxed area. (**B**) UMAP visualization of the expression levels of vascular SMC marker genes with the cell clouds containing vascular mural cells highlighted by the boxed area. (**C**) ISH for *Pdgfrb*, *Ephx3*, and *Pecam1* on a liver tissue section showing a region of a central vein. (**D**) IF for αSMA, CD31, CNN1, and NCAM1 on a liver tissue section, showing a region of a central vein (upper panel), portal and central vein (middle panel), or portal vein (lower panel). (**E**) Calculation of fibroblast or mural cell gene-score for the 90-gene list reported before (Muhl et al, 2020), for community clusters containing HSC and vascular mural cell types (stacked barplot showing mean values, for visualization fibroblast-gene expression values are inverted [multiplication by -1], error bars show s.d., *n* refers to cells: m3c = 113, m7c = 72, m5c = 87, m9c = 39, m8c = 72, m15c = 33, m2c = 236). (**F**) UMAP visualization of the expression levels of the pericyte marker genes (*Cspg4* and *Higd1b*), and *Pdgfra* in the mesenchymal cell dataset. (**G**) IF for CD31, ACE2, and αSMA on a liver tissue section from a $Pdgfrb^{GFP}$ reporter mouse. Arrows indicate pericytes at the peribiliary vasculature. PV portal vein, BD bile duct, HA hepatic artery, CV central vein. Scale bars are indicated in the respective image panels.

 © The Author(s)

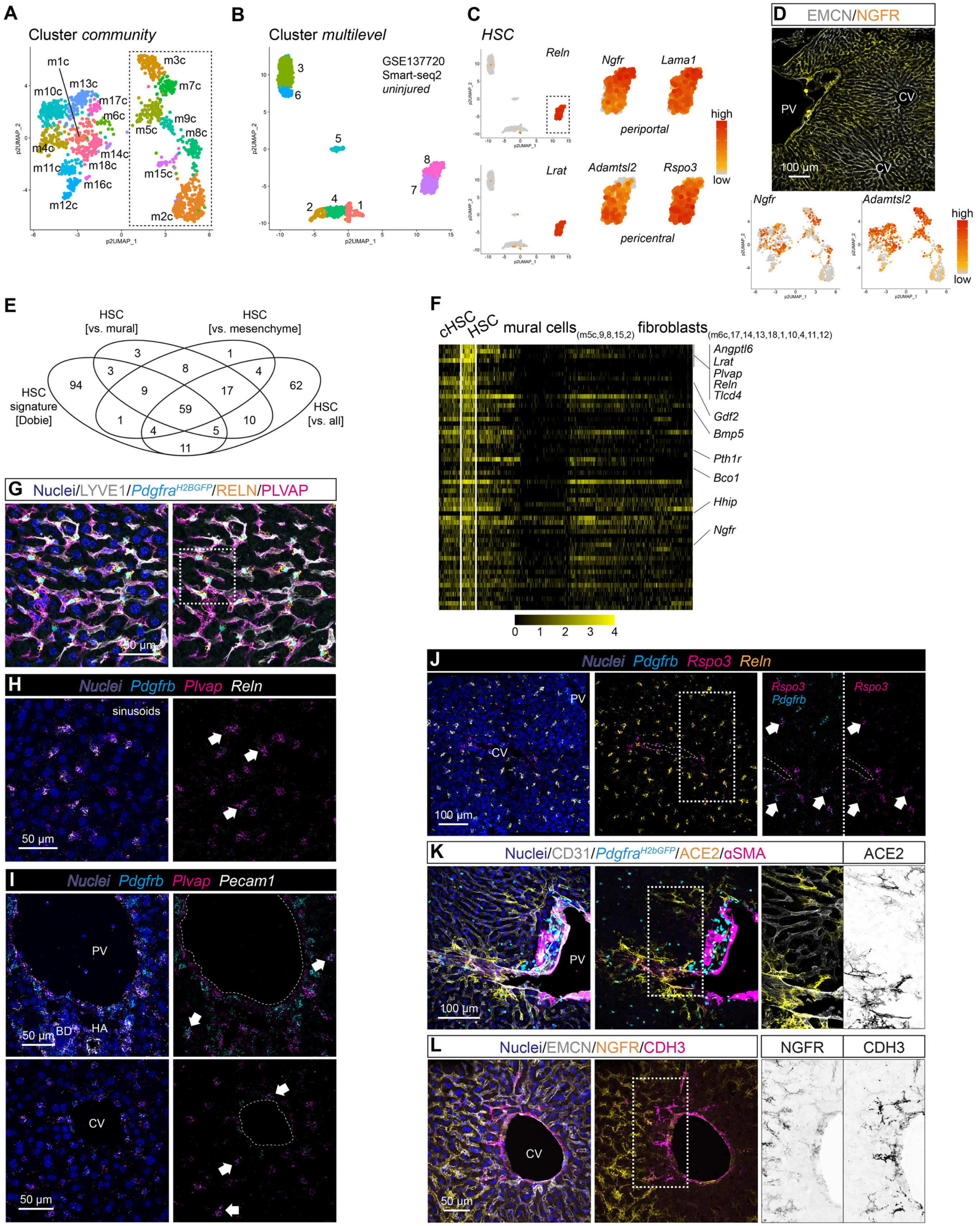
A
Cluster community
B
Cluster multilevel
GSE137720
Smart-seq2
uninjured
C
HSC
Reln
Ngfr
Lama1
periportal
Lrat
Adamtsl2
Rspo3
pericentral
high
low
D
EMCN/NGFR
PV
CV
100 µm
E
HSC [vs. mural]
HSC [vs. mesenchyme]
HSC signature [Dobie]
HSC [vs. all]
F
cHSC
HSC
mural cells(m5c,9,8,15,2)
fibroblasts(m6c,17,14,13,18,1,10,4,11,12)
Angptl6
Lrat
Plvap
Reln
Tlcd4
Gdf2
Bmp5
Pth1r
Bco1
Hhip
Ngfr
G
Nuclei/LYVE1/PdgfraH2BGFP/RELN/PLVAP
H
Nuclei Pdgfrb Plvap Reln
sinusoids
50 µm
I
Nuclei Pdgfrb Plvap Pecam1
BD
HA
J
Nuclei Pdgfrb Rspo3 Reln
K
Nuclei/CD31/PdgfraH2bGFP/ACE2/αSMA
ACE2
L
Nuclei/EMCN/NGFR/CDH3
NGFR
CDH3

◀ **Figure EV5. Analysis of HSC signature genes and marker gene expression.**

(**A**) UMAP visualization of the clustering result of the mesenchymal cell dataset using the community high resolution setting with HSC and vascular mural cell clusters indicated by the boxed area. (**B**) UMAP visualization of the clustering results of the GSE137720 (Smart-seq2) dataset using cells from uninjured samples. (**C**) UMAP visualization of the expression level of HSC marker genes (*Reln* and *Lrat*) and HSC zonation markers (*Ngfr*, *Lama1*, *Adamtsl2*, and *Rspo3*) magnified from the indicated area of the GSE137720 dataset. (**D**) IF for EMCN and NGFR on a liver tissue section (upper panel) and UMAP visualization of the expression levels of *Ngfr* and *Adamtsl2* in the mesenchymal cell dataset (lower panel). (**E**) Venn diagram showing the overlap of HSC-enriched genes calculated from the GSE137720 dataset using uninjured cells (Dobie), the mesenchymal cell subset (vs. mural, vs. mesenchymal), and the complete dataset (vs. all) (see also Dataset EV1). Note that 59 genes are commonly detected as enriched in HSC (cluster #m7c) in all four comparisons. (**F**) Heat map showing the expression levels of the 59 HSC enriched genes in the mesenchymal cell subset. (**G**) IF for LYVE1, RELN, and PLVAP on a liver tissue section from a *Pdgfra*^{H2bGFP} reporter mouse. The indicated boxed area is shown magnified in Fig. 7F. (**H**) ISH for *Pdgfrb*, *Plvap*, and *Reln*, on a liver tissue section focusing on the sinusoidal region. Arrows indicate *Pdgfrb Plvap* double-positive HSC. (**I**) ISH for *Pdgfrb*, *Plvap*, and *Pecam1* on a liver tissue section focusing on the portal tract (upper panel) or central vein (lower panel) region. Arrows indicate *Pdgfb Plvap* double-positive HSC. (**J**) ISH for *Pdgfrb*, *Rspo3*, and *Reln* on a liver tissue section. Arrows indicate *Pdgfrb Rspo3* double-positive HSC close to the central vein. (**K**) IF for CD31, ACE2, and αSMA on a liver tissue section from a *Pdgfra*^{H2bGFP} reporter mouse focusing on the portal tract. (**L**) IF for EMCN, NGFR, and CDH3 on a liver tissue section focusing on the central vein. PV portal vein, BD bile duct, HA hepatic artery, CV central vein. Scale bars are indicated in the respective image panels.

 © The Author(s)
